# Phytotherapeutics self-microemulsifying systems in pellet dosage form for enhanced intestinal drug delivery: formulation, stability, and *in-vivo* performance

**DOI:** 10.1080/10717544.2026.2702133

**Published:** 2026-07-15

**Authors:** Gabriela Koutná, Jan Kotouček, Jan Macků, Kateřina Kubová, Martina Urbanová, Larisa Janisová, Ivana Šeděnková, Jan Muselík, Jakub Vysloužil, Josef Mašek, Eliška Mašková, Miroslava Pavelková, David Vetchý, Jiří Brus

**Affiliations:** a Department of Pharmaceutical Technology, Faculty of Pharmacy, Masaryk University Brno, Brno, Czech Republic; b Department of Pharmacology and Toxicology, Veterinary Research Institute, Brno, Czech Republic; c Department of Structural Analysis, Institute of Macromolecular Chemistry, Czech Academy of Sciences, Praque, Czech Republic

**Keywords:** Enteric capsules, intestinal drug delivery, self-microemulsifying drug delivery system, pellets, volatile phytotherapeutics

## Abstract

Self-microemulsifying drug delivery systems (SMEDDS) containing volatile phytotherapeutics such as thymol (T), carvacrol (C), and eugenol (E) present significant formulation challenges, even when solidified. Their instability and interactions with coatings often hinder intestinal delivery. To address these limitations, we developed solid SMEDDS consisting of pellets (microcrystalline cellulose/magnesium aluminometasilicate/chitosan) and enteric capsules (CEC) for enhanced intestinal delivery. Based on solubility and pseudo-ternary phase diagrams, SMEDDS formulations (SES1-3) differing in component ratios (glycerol monooleate/caprylocaproyl macrogol-8 glycerides/diethylene glycol monoethyl ether) with 5% w/w of each drug were identified, demonstrating nano-scale droplet sizes (PDI <0.4) and showing no phase separation over 6 months. Thermodynamic stability and liquid-state NMR revealed particle size variations with preserved structural integrity. The lead formulation SES1 exhibited superior ex-vivo intestinal permeation (T-SES1). CECs filled with T-, C-, and E-loaded SES1 pellets, respectively, prepared via extrusion/spheronization, exhibited in-vitro gastro-resistant release, and achieved > 85% drug release within 120  min after a pH change to 6.8 during a one-year stability study (25 °C; 60% RH). FTIR-ATR analysis of the CEC internal surface confirmed the temperature-dependent restructuring of hypromellose and E sorption, a phenomenon not observed with C or T, which is likely attributable to physicochemical distinctions. Oral administration of CEC with T-SES1-pellets (0.5  mg/kg) in piglets demonstrated a delayed peak plasma concentration (C_max_ 11.67  ng/mL at 9 h) and sustained systemic exposure (AUC 119.8 ng·h/mL). These in-vivo findings substantiate the gastro-protective effect and enhanced intestinal absorption, positioning the pellet/CEC system as a promising strategy for the application of volatile phytotherapeutics in current pharmacotherapy.

## Graphical abstract

**Figure:**
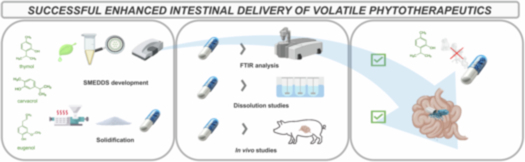

## 1. Introduction

Over the last few decades, natural active ingredients have gained extensive interest across various scientific disciplines, including pharmaceuticals, cosmetics, and the food industry. Many of these ingredients have been classified by the FDA as Generally Recognized as Safe and Effective (GRASE), highlighting their safety profiles and a broad range of biological activities (Souza et al. 2022). Among these GRASE compounds, monoterpenoid alcohols, such as thymol and its optical isomer carvacrol, exhibit strong antimicrobial, anti-inflammatory, antioxidant, antifungal, immunomodulant, and gastroprotective properties (Yanishlieva et al. 1999; De Santana Souza et al. 2017; Escobar et al. 2020; Mączka et al. 2023). Similarly, the well-studied phenolic compound eugenol, with its multidirectional actions and potent antioxidant and anti-inflammatory activity, is also a suitable candidate for the prevention and treatment of many diseases (Gülçin et al., 2011; Ulanowska and Olas 2021). Studies have shown that these natural active ingredients can be used alone or combined with other natural substances or other with compounds, such as antibiotics, providing a synergistic effect (Mączka et al. 2023; Gan et al. 2023).

Crohn's disease and ulcer colitis are the most common types of inflammatory bowel diseases (IBD), characterized by chronic inflammation of the gastrointestinal tract (GIT), leading to symptoms such as abdominal pain, diarrhea, rectal bleeding, and perianal lesions. The increasing prevalence and progressive nature of IBD strongly impact patients' quality of life (Roda et al. 2020). Currently, no cure exists (Yeshi et al. 2020). Available therapies, including 5-aminosalicylic acids, corticosteroids, immunomodulators, biologics, and surgical intervention for damaged gastrointestinal segments (Vieujean et al. 2025), aim to reduce inflammation, maintain remission of symptoms, prevent and treat complications, and promote mucosal healing (Cai et al. 2021). However, these treatments often provide limited benefits and are associated with significant risks, side effects, and severe constraints. Implementing new, safer, and more efficient compounds is required to ameliorate current therapeutic options and their outcomes, sustain remission, and potentially prevent disease progression. Phytotherapeutics, with their diverse health-promoting properties, hold promise as functional dietary supplements that could contribute to the prevention and remission of IBD, offering a complementary approach to existing therapies. Studies indicate that thymol promotes mucus secretion, enhances the intestinal barrier integrity, and combats invasive pathogens (Liu et al. 2022). Carvacrol reduces inflammatory biomarkers, restores tissue in the small intestine and colon (Imran et al. 2022), and exhibits gastroprotective properties (Oliveira et al. 2012). Combined, these monoterpenes significantly decrease intestinal oxidative stress (Wei et al. 2017). Additionally, eugenol reduces the disease activity index by attenuating oxidative stress and colonic inflammation (Chen et al. 2021).

Despite these benefits, the hydrophobic origin and volatility of these phytotherapeutics present a major challenge in formulation development. The volatility of these substances is influenced by their low molecular weight (150.22 g/mol; 150.2 g/mol; and 164.20 g/mol), vapor pressure (0.02 hPa, 0.05 hPa; and 0.03 hPa), and boiling point (232 °C; 238 °C; and 254 °C) for thymol, carvacrol, and eugenol, respectively (Weschler and Nazaroff 2008; Maffei et al. 2011). According to the Biopharmaceutical Classification System (BCS), thymol, carvacrol, and eugenol belong to Class II drugs, characterized by low solubility and high permeability. Therefore, to maximize its absorption potential, enhancement of the dissolution rates is required (Alqahtani et al. 2021). Many strategies have been applied to increase the solubility and the bioavailability of highly lipophilic drug compounds. Structural modifications, including salt and prodrug formation, particle micronization, or drug carrier systems formulation, such as cyclodextrins, micelles, and lipid-based formulations (LBFs), are only a few techniques for solubility modification (Franc et al.^2012^; Kumar et al. 2013; Li et al. 2018; Alqahtani et al. 2021). LBF has been used to facilitate drug absorption of BCS Class II and IV drugs, including the phytotherapeutics mentioned above. Based on their physicochemical properties, LBF can be further classified as lipid solutions, dispersed systems, nanoparticles, and liposomes. Pouton (Pouton 1997) introduced a Lipid Formulation Classification System that categorizes drug delivery formulations into Types I-IV.

Self-microemulsifying drug delivery systems (SMEDDS), Pouton's type III formulations (Pouton 1997; Seiberova et al. 2014), are defined as isotropic mixtures of oils and surfactants, or alternatively, cosurfactants/cosolvents. Upon dilution and gentle agitation in aqueous media, for instance, gastrointestinal fluids, these systems form an oil-in-water (o/w) emulsion with oil phase droplets between 100 and 300 nm or a microemulsion with a size of 50  nm (Pouton 2000; Pouton and Porter 2008). Currently, only a few products on the market utilize liquid self-emulsifying drug delivery systems (SEDDS) as carriers for active compounds, typically filled into soft gelatine (Salawi 2022) or hard gelatine capsules (Meirinho et al. 2022). While capsule filling is one of the simplest methods for encapsulating liquid SEDDS, it has several drawbacks, including interactions with the capsule shell, leakage, and handling difficulties. Solidification techniques, such as wet granulation, spray drying, or pelletization, address these issues by offering improved storage stability, precise dosing, and lower manufacturing costs (Mandić et al. 2017). Additionally, these methods provide significant advantages for encapsulating volatile compounds, such as terpenoids, which, like most essential oils, are sensitive to light, oxygen, and temperature changes, making them prone to oxidation or hydrolysis (Turek and Stintzing 2013). By reducing evaporation, minimizing surface area exposure, and offering protection against oxidation, solidification techniques preserve the stability and functionality of these volatile components (Ma et al. 2016). Among these approaches, solidifying liquid SEDDS into a pelletized form through extrusion/spheronization offers a particularly effective solution, combining the benefits of liquid SEDDS' self-emulsifying properties with the stability, ease of handling, and enhanced protection provided by the solid pellet formulation.

Last but not least, effective intestinal delivery of volatile phytotherapeutics remains a significant challenge owing to their partial absorption in the stomach. Addressing these GIT limitations could substantially enhance the therapeutic applications of these compounds. A recent study has demonstrated that gastro-resistant coatings for self-emulsifying pellets using commercially available Eudragit® L 30 D-55 exhibit strong interactions with SEDDS, leading to compromised gastro-resistance over extended periods (Macku et al. 2024). As a potential alternative, encapsulating volatile phytotherapeutics within solidified particulate systems inside enteric-coated hard capsules offers a simple and elegant solution to enhance their intestinal delivery. The newly marketed Capsugel® Enprotect® ready-to-fill capsules provide an innovative solution for delivering drugs to the small intestine. Composed of hypromellose (HPMC) and HPMC acetate succinate (HPMC-AS), they have been successfully validated for their protective capability against gastric conditions (Grimm et al. 2023). However, to date, no studies have investigated the interaction risks between volatile compounds encapsulated in solidified SEDDS formulations and commercially available enteric capsules.

This work aimed to address the challenge of intestinal delivery of SEDDS for volatile monoterpenoid phytotherapeutics. The experimental study is centered on the development of SMEDDS containing thymol, carvacrol, and eugenol, respectively, designed for targeted intestinal release via innovative pellet dosage forms incorporated into commercially available gastro-resistant capsules while thoroughly investigating their stability and *in-vivo* performance.

## 2. Materials and methods

### 2.1. Materials

Substances for SMEDDS formulations such as Peceol™ (Glycerol monooleate, Cat. No: 3088), Maisine® CC (Glycerol monolinoleate, Cat. No: 3431), Labrasol® ALF (Caprylocaproyl macrogol-8 glycerides, Cat. No: 3405), Labrafil® M 1944 (Oleoyl macrogol-6 glycerides, Cat. No: 3063), Capryol® 90 (Propylene glycol monocaprylate, Cat. No: 3254), and Transcutol® HP (Diethylene glycol monoethyl ether, Cat. No: 3265) were sourced as free samples from Gattefossé, France. Medium-chain triglycerides (Cat. No: 615780), and ethanol 96% (Cat. No: 605455) were purchased from Fagron, Czech Republic, and propylene glycol (Cat. No: 03000917) from Dr. Kulich Pharma, Czech Republic. The active pharmaceutical ingredients carvacrol (Cat. No: 282197) and eugenol (Cat. No: 1366759) were purchased from Sigma-Aldrich, Germany. Thymol (Cat. No: 03001031) was purchased from Dr. Kulich Pharma, Czech Republic. Milli-Q water was used to evaluate the formed microemulsions. Magnesium aluminometasilicate (Neusilin® US2; Fuji Chemicals, Japan, Cat. No: 297777), chitosan (JBICHEM, China, Cat. No: N/A), and microcrystalline cellulose (MCC) (Avicel® PH 101, FMC, Ireland, Cat. No: 11365) were used as powder excipients for the self-emulsifying pellets formulation and Capsugel® Enprotect® capsules (Lonza, Belgium, Cat. No: N/A) were employed as a carrier system for their enteric delivery.

### 2.2. Phytoterapeutics solubility studies

Various oily vehicles [medium-chain triglycerides (29 mPa.s); glycerol monooleate (220 mPa.s); glycerol monolinoleate (120 mPa.s)], surfactants [caprylocaproyl macrogol-8 glycerides (80–110 mPa.s); oleoyl macrogol-6 glycerides (75–95 mPa.s); propylene glycol monocaprylate (20 mPa.s)], and cosurfactants/cosolvents [diethylene glycol monoethyl ether (4.8 mPa.s); propylene-glycol (52 mPa.s); ethanol 96% (1.2 mPa.s)] were selected to screen the solubilization capacity of testing media. The solubility studies were performed by adding excessive amounts of each natural drug (2,500 mg) into 15 mL Falcon tubes containing 2 mL of the selected solvents. The samples were mixed for 2 min using a vortex mixer (Vortex, Allpax, Germany), followed by continuous mixing of the samples on a roller mixer (SRT9, Stuart, Germany) for 24  h at room temperature (Schmied et al. 2022). Subsequently, samples were withdrawn, centrifuged for 30 min (400 × g, Eppendorf centrifuge, Germany), and filtered using a membrane filter (0.45  µm, Millipore). Aliquots of the supernatant were diluted with methanol, and the drug concentration was quantified using the validated HPLC method as described in Chapter 2.8.

### 2.3. Optimization of SMEDDS components ratio and sample designation

Ten formulations composed of the selected ingredients glycerol monooleate, caprylocaproyl macrogol-8 glycerides, and diethylene glycol monoethyl ether in varying ratios were labeled as SES1 through SES10 (Table S1, *Supplementary Material*). S_mix_ refers to the mixture of caprylocaproyl macrogol-8 glycerides and diethylene glycol monoethyl ether in varying ratios. Ten blank and ten drug-loaded (5% w/w) SMEDDS preconcentrates were prepared as an initial formulation screening step to evaluate the miscibility and physical stability of the selected excipients. To form 500 mg of SMEDDS preconcentrates, glycerol monooleate, caprylocaproyl macrogol-8 glycerides, and diethylene glycol monoethyl ether were mixed in desired ratios, vortexed for 2 min, and diluted with 500  μL Milli-Q water (1:1 ratio). Samples were visually assessed immediately after dilution and after 24 h at room temperature for macroscopic homogeneity, phase separation, and drug precipitation. Formulations showing no visible instability during the observation period were considered suitable for further development and subjected to subsequent quantitative characterization. Selected formulations were labeled as *X*_SES1, *X*_SES2, and *X*_SES3, where ‘*X*’ represents the active compound in the system (T for thymol, C for carvacrol, E for eugenol).

### 2.4. Pseudo-ternary and ternary phase diagrams study

Pseudo-ternary phase diagrams of oil, surfactant, cosurfactant, and water were plotted to identify the microemulsion region using a water titration method. At first, S_mix_ systems were prepared by mixing surfactant (caprylocaproyl macrogol-8 glycerides) and cosurfactant (diethylene glycol monoethyl ether) in fixed ratios of 1:4, 1:3, and 1:2. For each phase diagram, oil and selected S_mix_ ratios were mixed thoroughly in ratios 1:9, 2:8, 3:7, 4:6, 5:5, 6:4, 7:3, 8:2, and 9:1 to obtain 1000  mg of SMEDDS preconcentrate and were vortexed until homogeneous solution was obtained. All formulas were titrated with purified water at room temperature and were visually observed for phase clarity. Once the emulsions turned turbid, the weight of added water was noted. The pseudo-ternary phase diagrams were drawn by the Ternary Plot online generator. As described before, ternary diagrams of drug-loaded SMEDDS (5% w/w) were plotted as well to recognize the changes in the microemulsion region after adding active ingredients.

### 2.5. Microemulsion systems − droplet size analysis, emulsification properties, long-term stability studies, and confocal laser scanning microscopy

The mean hydrodynamic diameter (Z-Ave), size distribution, and zeta-potential of prepared microemulsions were assessed using a Zetasizer (Nano ZS, Malvern Instruments Ltd, UK). Particle size and size distribution were measured via the Dynamic Light Scattering (DLS) measurement at 25 °C employing non-invasive backscattered light detection at 173°. Samples were diluted with purified water and measured in disposable polystyrene cuvettes. Each sample was measured in triplicate. Selected formulations in the amount of 100 mg were progressively titrated with 10% w/w of purified water up to the point when turbidity was reached. After each water addition, samples were vortexed for 30 s and placed into the module. Each sample was measured in triplicate. The polydispersity index (PDI) was used to illustrate the size distribution, and the average particle size (Z-Ave) was reported as the intensity mean diameter. Zeta potential was determined by measuring the velocity of particle movement using Doppler laser electrophoresis after 60% w/w sample dilution with purified water.

All SMEDDS formulations underwent long-term stability tests at the time of preparation, after 1, 2, 3, and 6 months. During this period, samples were stored at room temperature ± 25 °C and at a refrigerator temperature of 2−8 °C. Samples were diluted with 60% w/w water before each assessment. Droplet size analysis and PDI measurements were carried out for all formulations.

The time it takes for a formulation to form a homogeneous emulsion was evaluated by a standard USP dissolution apparatus (Sotax AT-7 Smart, Sotax, Switzerland). Each formulation (5 mL) was added to 250  mL of purified water heated up to 37 ± 0.5 °C with a paddle rotation of 50  rpm. The appearance and emulsification speed were assessed using a well-known grading system (Van Staden et al. 2020).

Moreover, the physicochemical stability and homogeneity of the selected T-loaded emulsions were visualized using confocal laser scanning microscopy (Leica SP8, Germany). Each SMEDDS preconcentrate, T_SES1, T_SES2, and T_SES3 (1000  mg), was mixed with 20  μL of green fluorescent, lipophilic carbocyanine probe DiOC_18_. Stained SMEDDS formulations were diluted with Milli-Q water in a 1:1 ratio and briefly vortexed. A White Light laser was used for excitation (488 nm), and the emission spectrum was observed at 505–550 nm. Particle-associated fluorescent domains were analyzed using ImageJ software. Images were converted to 8-bit format, thresholded, and analyzed using the Analyze Particles function after scale calibration. Feret diameter was used to estimate the apparent particle size distribution.

### 2.6. Thermodynamic stability and liquid-state NMR studies of SMEDDS formulations

Selected SMEDDS samples were exposed to thermodynamic stability testing (heating–cooling cycle, centrifugation, and freeze–thaw cycle) following previously established protocol with minor adjustments (Shafiq et al. 2007). Six 48-h cycles between refrigerator temperatures of 4 °C and 45 °C were carried out. Samples showing no phase separation, drug precipitation, or creaming were subjected to a centrifugation test, where the formulations were centrifuged at 3500 rpm for 30 min (Eppendorf™ Centrifuge 5810/5810 R, Eppendorf, Germany). Formulations showing no phase separation were further subjected to the freeze-thaw cycle with a temperature varying between −21 °C and +25 °C. After withdrawal, samples were observed for drug precipitation, phase separation, or creaming. Droplet size analysis and PDI measurements were conducted to observe if any changes occurred after thermodynamic stability tests.

High-resolution ^1^H NMR spectra were recorded using a Bruker Avance III 600 spectrometer operating at 600.2  MHz (Bruker BioSpin, Germany). Samples were filled into 5 mm NMR tubes. To adjust the NMR experimental parameters, an NMR capillary tube filled with deuterated D_2_O was used. Each experiment was performed with 18  μs ^1^H NMR 90° pulse, an acquisition time of 2.1 s, 32 scans, and 10 s relaxation delay between scans. In all experiments, the temperature was kept constant within 0.2 K using a BVT 3000 temperature unit.

### 2.7. *Ex-vivo* passive permeation into the intestinal epithelium

The diffusion rate through the swine intestine was assessed in the selected samples, T_SES1, T_SES2, and T_SES3, using Franz diffusion cells. SMEDDS formulations loaded with thymol were selected as the representative samples. Intestinal samples were obtained following the 3Rs (Replacement, Reduction, and Refinement) principle (Madden et al. 2012). The proximal part of the swine intestine was excised and thoroughly cleaned. The tissue was obtained from a piglet euthanized at the conclusion of a prior experiment approved by the Ministry of Agriculture Ethics Committee, Czech Republic (approval number MZe 2581). The receptor cell was filled with a 50% methanol solution and included a small magnetic stirrer to maintain homogeneity during the experiments. The intestine was positioned between the receptor and donor cells, with the basal side in contact with the receiving medium and the apical side in contact with the donor chamber. Care was taken to prevent bubble formation, and the intestine was securely held in place using metal clamps. The donor cell was then loaded with 2000 µL of the SMEDDS sample. The diffusion area of the receptor cells was 7.06  cm^2^. The temperature was maintained at 37 °C throughout the experiments, and the samples were continuously stirred at 350 rpm to ensure uniformity. Sampling was performed at specified time intervals (30, 60, 90, 120, 180, 240, and 300 min) over 5 h, with 500 µL samples collected from the middle of the receptor compartment at each time point. The removed sample was promptly replaced with fresh medium (50% methanol). While this approach is an accepted strategy for lipophilic permeants (Marques-da-Silva et al. 2025), it is acknowledged that the non-physiological receptor medium composition may overestimate absolute permeation rates. Accordingly, the present study was intended primarily for comparative evaluation among formulations rather than direct prediction of *in-vivo* permeability. Sample analyses were performed using liquid chromatography-tandem mass spectrometry. A triple quadrupole mass spectrometer Agilent 6410 Triple Quad LC/MS (Agilent Technologies, USA) with electrospray ionization (ESI) was used to detect the analyte. An Agilent 1200 chromatographic system (Agilent Technologies, Germany), consisting of a binary pump, vacuum degasser, autosampler, UV detector, and thermostated column compartment, was used.

### 2.8. Formulation and evaluation of self-emulsifying pellets, Enprotect® capsules filling

The formulation X_SES1, loaded with thymol, carvacrol, and eugenol (5% w/w), respectively, was selected for solidification into a pellet dosage form following a previously established method (Tan et al. 2013; Macku et al. 2022). Briefly, powder, well-established and safe, ingredients (MCC – 50 g, magnesium aluminometasilicate – 30 g, and chitosan 20 g) were mixed and homogenized at 1500 rpm in a vertical homogenizer Stephan UMC 5 (A. Stephan u. Söhne GmbH & Co., Germany). Subsequently, the SMEDDS mixture (60 g) containing 3.0 g of the respective phytoterapeutic and water (75 g) was stepwise added to the powder mixture under constant stirring to ensure a high degree of homogenization of the matrix. The extrusion/spheronization parameters applied in this study were selected based on preliminary formulation development experiments aimed at obtaining processable wet masses and spherical pellets, rather than through systematic optimization of critical process parameters. The wetted matrix was extruded using an axial screw extruder (Pharmex 35T, Wyss & Probst, Ettlingen, Germany) equipped with a die plate featuring 0.78 mm perforations, operating at a constant speed of 110  rpm. The extrudate was manually transferred onto the 23 cm radial plate of the spheronizer (Pharmex 35T, Wyss & Probst, Germany) and spheronized at 1000 rpm for 2 minutes. The resulting pellets were dried on trays in a hot-air dryer (Type 38A, Horo, Germany) at 40 °C for 12 h and marked as *P*-T_ SES1, *P*-C_ SES1, and *P*-E_ SES1 (‘P’ = pellets).

The obtained pellets were size-fractionated using a sieve shaker and stainless-steel mesh sieves (125–2000 µm). The fraction 0.5–0.8 mm was used for the evaluation of flow properties, Hausner ratio, sphericity, drug content, and *in vitro* dissolution studies. The equivalent diameter and sphericity (S) of the carrier pellets were determined through image analysis. For each batch of every sample, 200 pellets were randomly selected and analyzed using a NIKON SMZ 1500 stereoscopic microscope (Nikon, Japan), equipped with a 72AUC02 USB camera (The Imaging Source, Germany). Image acquisition and processing were conducted using NIS-Elements AR 4.0 software (Nikon, Japan). The equivalent diameter was computed based on the projected area of each pellet, under the assumption of a spherical geometry. Sphericity was subsequently calculated using the following equation:

$$S=\;\frac{4\pi A}{p^{2}}$$

where **S** is sphericity, **A** is surface area (mm^2^), and **p** is perimeter (mm).

The Hausner ratio (HR), indicating pellet flow properties, was evaluated in triplicate (Ph. Eur. 10). The pycnometric density of the pellet samples (*n* = 3) was measured using a helium pycnometer (Pycnomatic-ATC, Porotec GmbH, Haan, Germany).

Subsequently, enteric capsules filled with pellets (420  mg) were stored at 25 °C, 60% RH, and at 40 °C, 75% RH for stability studies. To determine the content of thymol, carvacrol, and eugenol in pellets, 100  mg of pellets (*n* = 3) were crushed and placed into a volumetric flask, and 50  mL of distilled water was added. Flasks were ultrasonicated to support drug release from the dosage form for 10 min (Bandelin Sonorex, Germany). Afterward, methanol was added up to a total volume of 100 mL, and samples were incubated for 24 h at room temperature. Samples were filtered through a 0.45 μm membrane pore filter, and 10  μL of each sample was analyzed for drug content via HPLC using LiChrospher® 100 RP-18 column (particle size 5 μm). The mobile phase consisted of 50% acetonitrile and 50% phosphoric acid (0.02 M), with a flow rate of 1.0 mL/min, and the column temperature was maintained at 30 °C. Spectra were recorded at a wavelength of 274 nm. Drug quantification was based on the linearized calibration curve (R^2^ ≥ 0.99). Validation was performed as follows. Calibration curves were constructed by plotting the peak area ratio against analyte concentration. The method exhibited linearity over the concentration range of 0.005–0.05 mg/mL, with a coefficient of determination (R^2^) greater than 0.99. Interday precision was assessed by analyzing calibration samples on different days, with one replicate measured per day. Intraday precision was evaluated through repeated analyses of calibration samples within a single day. Precision was expressed as the relative standard deviation (RSD, %). Interday and intraday precision ranged from 0.83–1.62% to 0.20–0.75%, respectively, including values ​​for all drugs. Accuracy was determined by comparing the measured concentrations of thymol, carvacrol, and eugenol with their nominal concentrations at three concentration levels. The accuracy, expressed as percent recovery, was ≥98%. Selectivity was demonstrated by comparing chromatograms of the excipient solution mixture (blank sample) with those of solutions containing thymol, carvacrol, and eugenol. The limit of quantification (LOQ) was determined using the signal-to-noise (S/N) ratio approach. Measured responses of blank samples were compared with those of samples containing known low concentrations of the analyte to establish the minimum concentration for reliable quantification. Based on an S/N threshold of 10:1, the LOQ was established at 0.65  µg mL^−1^ for both thymol and eugenol, and at 0.62  µg mL^−1^ for carvacrol.

For the *in vitro* dissolution studies (Sotax AT-7 Smart, Sotax, Switzerland), 420  mg pellets were filled into enteric capsules (*n* = 3) (marked as CP-T_ SES1, CP-C_ SES1, and CP-E_ SES1; ‘C’ = capsule) to deliver the pellets to the small intestine and protect the formulation against the harsh stomach environment (Grimm et al. 2023). To mimic the conditions of GIT fluids, artificial gastric juice without pepsin at pH 1.2 (1 g of NaCl and 2.85  mL of 35% HCl in 500 mL aqueous solution) was used for the first two h, followed by a pH increase to 6.8 (small intestine) by adding Na_3_HPO_4_.12H_2_O (6.25 g/500 mL) (Macku et al. 2022) for the remaining testing time (4 h). Samples were withdrawn at specified time points (0, 60, 120, 135, 150, 180, 210, and 240  min) and analyzed by HPLC as described above.

### 2.9. Stability studies and FTIR analysis

Long-term stability studies (0, 3, 6, 9, and 12 months) were carried out to evaluate the phytotherapeutics content and *in vitro* dissolution of capsule samples CP-T_ SES1, CP-C_ SES1, and CP-E_ SES1 at storage conditions of 25 °C, 60% RH and at 0, 3, and 6 months studies were carried for the 40 °C, 75% RH storage conditions. The drug content and *in vitro* dissolution release were determined using the methods described above (Chapter 2.8).

At 3 and 6 months of the stability testing, the Fourier Transform Infrared Spectroscopy (FTIR) spectra of the inner surface of the capsules (CP-T_SES1, CP-C_SES1, and CP-E_SES1) stored under both storage conditions were analyzed. A small section of the capsule shell's inner surface, which was in direct contact with the pellets, was carefully cut and examined using attenuated total reflectance (ATR) mode with a diamond GoldenGate (Specac). The spectra were recorded with a resolution of 4  cm^−1^, and 256 scans were accumulated for each measurement and compared with the empty enteric capsules as a reference. The ATR technique allows the study of the surface properties of the capsule material, the sorption of the drug into the capsule shell, and any system changes occurring during storage.

### 2.10. In-vivo studies

A pharmacokinetic study was conducted using healthy piglets (*Sus scrofa domestica*, *n* = 5, mean body weight 24.8 kg ± 1.8 kg) with ethical permit ID: MZe 2422. The number of animals was determined based on feasibility and standard practice to obtain an initial descriptive assessment while minimizing animal use in accordance with the 3Rs. The animals were sourced from Bioprodukt Knapovec, a.s., Czech Republic, and were confirmed to be free of pre-existing medical conditions prior to the study commencement. A minimum acclimatization period of 7 days at the research facility preceded the initiation of the experiment (time zero). In adherence to ethical guidelines for animal welfare and acknowledging the constraints on animal numbers in preclinical research, only a selected subset of self-emulsifying pellets containing the SMEDDS formulation T_SES1 was employed for subsequent pharmacokinetic evaluation. Each animal received an oral administration of two filled enteric capsules, corresponding to an administered dose of 0.5 mg of thymol per kilogram of body weight. Blood samples were collected at 0, 1.5, 3, 4.5, 6, 7.5, 9, 10.5, 12, and 24 h post-administration. Plasma samples were immediately frozen and stored at –80 °C until analysis by liquid chromatography–tandem mass spectrometry. No animals, samples, or data points were excluded from the analyses; all collected data were included. All animals were handled under identical husbandry and experimental conditions, blood samples were collected at the same predefined time points for all animals, and the analytical evaluation of samples was performed blinded to sampling time.

At the planned endpoint, piglets were first anesthetized by a single intramuscular bolus of a TKXZ mixture targeting 2 mg/kg of each component: xylazine (Xylazin Ecuphar 20 mg/mL), ketamine (Narkamon 100 mg/mL), and tiletamine/zolazepam (Zoletil 100; Virbac; reconstituted per manufacturer’s instructions). Virbac; reconstituted per manufacturer’s instructions). After recumbency, an intravenous catheter was placed in the lateral auricular vein and the depth of anesthesia was verified (no response to ear, tail and/or interdigital pinch, reduced jaw and limb tone, absent palpebral reflex). If required, anesthesia was deepened with ketamine 0.5–1.0 mg/kg IV (Narkamon 100 mg/mL). Euthanasia was then performed by intravenous T61 (Intervet/MSD Animal Health) at 1 mL/10 kg body weight via the auricular catheter, followed by confirmation of death. All procedures were performed by trained personnel in accordance with institutional SOPs and applicable national/EU legislation for the use of animals in research.

### 2.11. LC–MS/MS analysis

A volume of 200 µL of plasma was deproteinized by the addition of 1000 µL of 2-propanol. After centrifugation at 13,500  rpm (225  Hz) for 15 minutes, the resulting supernatants were evaporated to dryness. The residues were then reconstituted in 160 µL of 80% (w/w) acetonitrile and injected onto a Poroshell 120 EC-C18 column (3.0 × 100 mm, Agilent Technologies, Santa Clara, CA, USA).

Chromatographic separation was achieved using a 19-min linear gradient (80%–100% w/w acetonitrile) at a flow rate of 0.3  mL/min. Detection was performed on an Agilent 6410 Triple Quadrupole LC/MS system (Agilent Technologies, Santa Clara, CA, USA) equipped with an electrospray ionization source operating in positive ion mode.

Instrument settings included a fragmentor voltage of 100 V, gas temperature of 200 °C, gas flow rate of 6 L/min, nebulizer pressure of 35 PSI (241,317 Pa), and a capillary voltage of 5000 V.

Analysis was carried out in multiple reaction monitoring (MRM) mode, with thymol quantified using the transition m/z 151 → 109 and confirmed with the transition m/z 151 → 119 at a collision energy of 18 V.

Pharmacokinetic parameters, including maximum plasma concentration (C_max_), time to reach maximum concentration (T_max_), and area under the plasma concentration-time curve (AUC), were calculated. The AUC (0–12) was determined by the trapezoidal rule. All pharmacokinetic analyses were performed using GraphPad Prism software, version 8.0 (GraphPad Software, San Diego, CA, USA).

## 3. Results and discussion

Addressing the challenge of intestinal delivery for volatile phytotherapeutics via SEDDS, this work presents an experimental study centering on the development of SMEDDS containing thymol, carvacrol, or eugenol, respectively, formulated into innovative pellet matrices within commercially available gastro-resistant capsules for targeted intestinal release. To achieve this goal, it was necessary to approach the issue at several levels related to the course of the entire formulation. Therefore, the experiment consisted of SMEDDS optimization, incorporation to pellets/capsule dosage form, stability study, and final *in–vivo* study, with each stage including a thorough testing.

### 3.1. Solubility studies and optimization of SMEDDS component ratios

Ensuring sufficient solvent capacity for active substances is critical for achieving stable SMEDDS and preventing drug precipitation over an extended shelf life (Macku et al. 2022). All tested media exhibited high solubility, with more than 600  mg/mL for each tested natural drug (Table S2, *Supplementary Material*). Therefore, the final excipients were selected based on their miscibility and compatibility. Following a preliminary screening of several SMEDDS variations for stability and compatibility, ten formulations (SES1–SES10) were selected for further in-depth analysis (Table S1, *Supplementary Material*).

Given that the solubilization capacity of lipophilic moieties increases with the chain length of the oily phase, glycerol monooleate, composed of long-chain fatty acids (C18:1), was selected as the oily vehicle (Date and Nagarsenker 2008). Caprylocaproyl macrogol-8 glycerides were chosen as the primary surfactant due to their high HLB value (12), which promotes efficient emulsification and the formation of stable oil-in-water (o/w) emulsions with small droplet sizes (Kommuru et al. 2001; Matsaridou et al. 2012). Its non-ionic nature also offers lower toxicity and reduced gastrointestinal irritation compared to ionic surfactants, making it a safer option for pharmaceutical formulation (Hsieh et al. 2023). Diethylene glycol monoethyl ether was chosen as a cosurfactant to aid in stabilizing the microemulsion by enhancing the fluidity of the surfactant film (Isailović et al. 2017).

### 3.2. Optimization of SMEDDS components ratios

Optimization of SMEDDS component ratios revealed that among the ten formulations tested (SES1–SES10; Table S3, *Supplementary Material*), the blank formulations (SES1, SES2, SES3, and SES4) consistently formed clear, stable microemulsions upon 1:1 water dilution and exhibited no phase separation or drug precipitation over 24 h at room temperature. In contrast, formulations SES5–SES10 did not maintain this stability. In the case of drug-loaded formulations (5% w/w), *X*_SES1, *X*_SES2, and *X*_SES3 (for *X*—T_thymol, C_carvacrol, and E_eugenol) were able to form clear, stable microemulsions over the same 24-h period. However, formulation *X*_SES4 showed phase separation upon drug incorporation, indicating a destabilization of the system. Consequently, the stable drug-loaded formulations X_SES1, X_SES2, and X_SES3 were selected for further evaluation. The specific composition of these selected formulations is detailed in Table 1.

**Table 1.** Composition of selected SMEDDS preconcentrates. “*X*” indicates a drug loaded into the formulation (T-thymol; C-carvacrol; E-eugenol). Values are displayed in percentage (w/w).

Formulation	Thymol/Carvacrol/Eugenol	Glycerol monooleate	Caprylocaproyl macrogol-8 glycerides	Diethylene glycol monoethyl ether
*X*_SES1	5.0	9.5	68.4	17.1
*X*_SES2	5.0	9.5	64.1	21.4
*X*_SES3	5.0	9.5	57.0	28.5

### 3.3. Pseudo-ternary and ternary phase diagrams

Pseudo-ternary and ternary phase diagrams enable the identification of regions where microemulsions can be formed (Berkman and Güleç 2021). The largest microemulsion regions were achieved in SES1 formulations (S_mix_ ratio of 4:1) (Figure S1; *Supplementary Material*). A reduction in the amount of caprylocaproyl macrogol-8 glycerides in the formulations with S_mix_ ratios of 3:1 (SES2) and 2:1 (SES3) resulted in smaller microemulsion regions. Comparing phase diagrams of formulations with and without the drug revealed a reduction in microemulsion regions when the active ingredient (5% w/w) was incorporated. Similar findings were reported in another study, where adding oleanolic acid (20  mg/mL) to SEDDS reduced the self-emulsifying region (Xi et al. 2009). Notably, samples with the lowest oil concentration could form a clear and stable microemulsion with purified water, even up to 90% w/w. However, increasing the oil phase beyond 20% while reducing S_mix_ concentrations heightened interfacial tension between the water and oil phases. This, in turn, lowered the system’s ability to incorporate water and form stable, transparent emulsions (Kallakunta et al. 2012). The microemulsion regions identified by visual turbidity assessment were subsequently supported by instrumental droplet size and PDI measurements across increasing water concentrations using the water titration method (Table S4; Supplementary Material), as detailed in the following Section 3.4.

### 3.4. Droplet size analysis, long-term stability studies, and confocal laser scanning microscopy

Droplet size analysis is crucial for optimal drug release, *in-vivo* absorption, and formulation stability (Salawi 2022). The water titration method was used to monitor changes in particle size with increasing water content (Table S4; *Supplementary Material*). At 20% (w/w) water concentration, the formulations remained highly viscous with poor emulsifying properties, resulting in a high particle size and PDI. In contrast, dispersions diluted to 30–60% (w/w) formed a stable, transparent microemulsion with droplet size below 370 nm and PDI under 0.4. Thymol and carvacrol formed SMEDDS with particle sizes ranging from 150 to 360 nm. The smallest particle sizes (<100 nm) were observed in the eugenol-loaded SMEDDS. It was found that eugenol, exhibiting its inherent surface activity, led to smaller droplet sizes and improved emulsion stabilization (Terjung et al. 2012; Suriyarak and Weiss 2014). Furthermore, SES1 generally exhibited smaller particles and lower PDI values compared to samples SES2 and SES3, attributed to a higher surfactant concentration (Zhuang et al. 2011). Nevertheless, this phenomenon was not observed for formulations loaded with eugenol, which further confirms eugenol's capability of reducing interfacial tension. An increase in the size and PDI for all formulations was observed at dilutions exceeding 70%, suggesting potential system destabilization. Zeta-potential measurements at 60% (w/w) dilution remained close to the zero net charge, with values between −10 mV and +10 mV considered neutral (Clogston and Patri 2011). This is consistent with steric rather than electrostatic stabilization, as the polyethylene glycol-8 chains of caprylocaproyl macrogol-8 glycerides form a hydrated steric barrier at the oil-water interface, preventing droplet coalescence independently of surface charge and thereby contributing to physical stability, in agreement with previously reported microemulsion systems formulated with the same surfactant (Mehanna et al. 2020).

**Figure 1.**
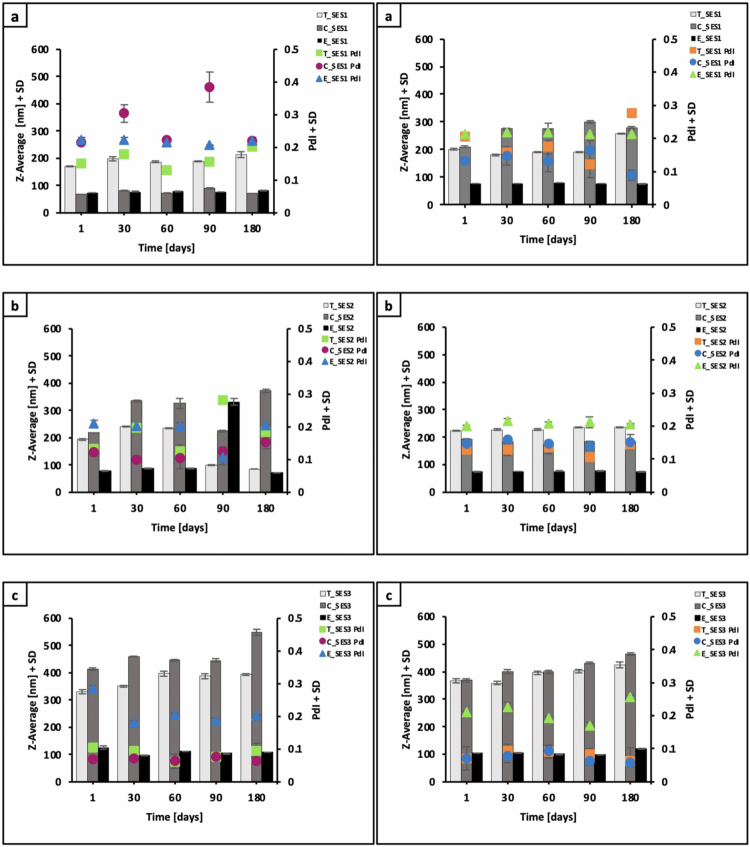
The SMEDDS long-term stability. The samples were stored in the fridge (left column) and at room temperature (right column) and were diluted with 60% (w/w) Milli-Q water prior to each assessment. (A) Stability of *X*_SES1 systems (T-thymol; C-carvacrol; E-eugenol); (B) Stability of *X*_SES2 systems (T-thymol; C-carvacrol; E-eugenol); (C) Stability of *X*_SES3 systems (T-thymol; C-carvacrol; E-eugenol).

SMEDDS long-term stability tests (Figure 1) showed no significant increase (>200 nm) in droplet size in any formulation after 60% (w/w) water dilution. In the T_SES1 formulation, particle size increased slightly by 70 nm at room temperature and 30 nm at 2–8 °C after 180 days. T_SES2 formulation exhibited a size reduction from 260 to 100 nm after 90 days at 2−8 °C, while T_SES3 formulation showed no changes in the particle size over 180 days. C_SES1 formulation formed an emulsion with a 4-fold lower particle size when being stored at 2−8 °C compared to storage at room temperature. Conversely, the C_SES2 formulation formed 2−fold bigger particles when being stored in the fridge than at RT. C_SES3 and formulations containing eugenol (E_SES1; E_SES2; E_SES3) remained stable under both storage conditions. The Z-average is an intensity-weighted mean that is particularly sensitive to the presence of even a small fraction of larger droplets; therefore, an increase in Z-average is best interpreted as an indicator of droplet growth and aggregation/coalescence rather than a uniform shift of the entire size population. Although some formulations exhibited size increases, the Đ of all formulations remained below 0.4, indicating an acceptable size distribution (Bhattacharjee 2016). Intensity-weighted particle size distribution plots are included in the *Supplementary Material* (Figure S2–S4). Although some formulations exhibited size increases, the PDI of all formulations remained below 0.4, indicating an acceptable, narrow size distribution (Bhattacharjee 2016). The results suggest that different storage conditions had an insignificant effect on the droplet size of the tested formulations.

**Figure 2.**
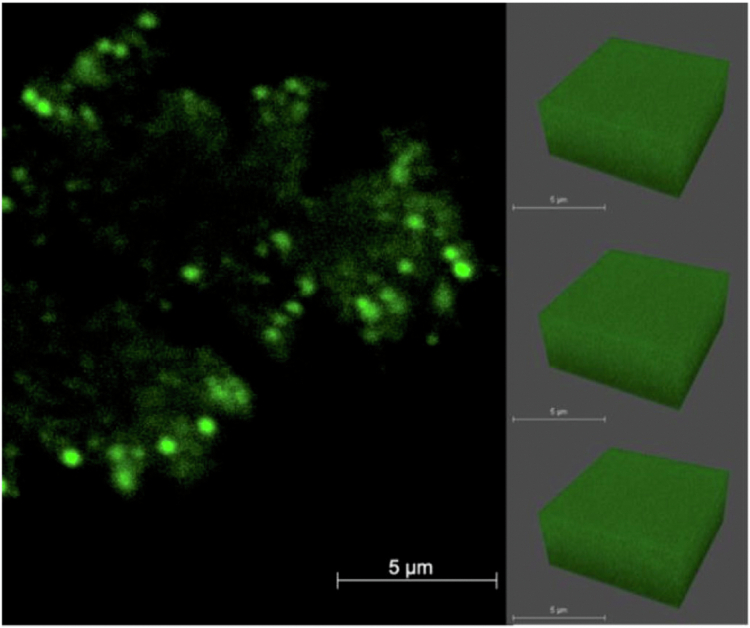
Detailed visualization of the emulsion formed by fluorescently labeled T_SES1 formulation after being dispersed in Milli-Q water (left). Visualization of T_SES1, T_SES2, and T_SES3 formulation loaded with fluorescent lipophilic dye, diluted with Milli-Q water in a 1:1 ratio (right).

To demonstrate the particle distribution and homogeneity of the prepared SMEDDS, thymol-loaded (T_SES1, T_SES2, and T_SES3) were additionally evaluated by confocal laser scanning microscopy with the green fluorescent dye DiOC_18_. All selected systems successfully formed homogeneous emulsions upon dilution with water (Figure 2, right). A detailed view of the oily droplets after the dispersion of the T_SES1 formulation demonstrated a relatively uniform fluorescent distribution throughout the sample (Figure 2, left). Subsequent ImageJ analysis of the confocal images revealed a heterogeneous fluorescent domain size distribution with a mean Feret diameter of 342 ± 85 nm (*n* = 161).

### 3.5. Thermodynamic stability test and liquid-state NMR studies

**Figure 3.**
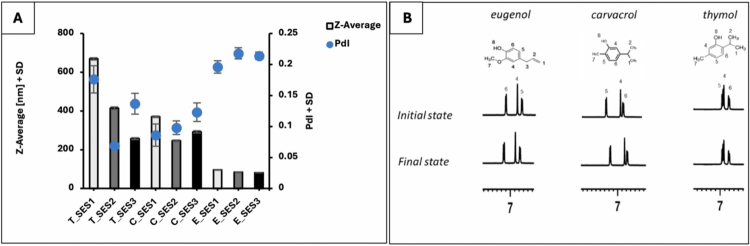
Thermodynamic stability evaluation. (A) Particle size and PdI measurements following thermodynamic stability tests. The samples were diluted with 60% w/w Milli-Q water. The indicated values are the means + SD. (B) ^1^H NMR spectra of used phytotherapeutics’ region for the initial and final state.

The tested formulations successfully withstood heating–cooling, centrifugation, and freeze–thaw cycles. Throughout these tests, no macroscopic alterations indicative of destabilization, such as drug precipitation, turbidity, or phase separation, were observed (Table S5, *Supplementary Material*). Droplet size measurements, conducted after a 60% dilution of each sample with Milli-Q water (Figure 3A), revealed notable increases in droplet size (>200 nm) for specific systems when compared to initial measurements (Day 1, Figure 1). Specifically, T_SES1 increased from approximately 200 to 670 nm, T_SES2 from 200 to 450 nm, and C_SES1 from approximately 69 to 370 nm. In contrast, systems containing eugenol maintained stable droplet sizes. This stability can be attributed to eugenol's previously reported ability to reduce interfacial tension and stabilize emulsions, even at low concentrations (Terjung et al. 2012). The PDI of each formulation remained below 0.3 following dilution, confirming the acceptable, narrow size distribution nature of the systems.

Changes in all investigated compounds were monitored after each cycle (heating-cooling, centrifugation, and freezing-thawing) using ^1^H NMR spectroscopy. Reversible changes in the position of caprylocaproyl macrogol-8 glycerides/glycerol monooleate signals are associated with the thermosensitivity of the individual components themselves and do not affect the drugs' behavior. No alterations were detected in the thymol, carvacrol, or eugenol regions of the spectra (Figure 3B). Overall, the samples remained stable throughout the cycles, exhibiting no changes from their initial state.

### 3.6. *Ex*-*vivo* passive permeation into the intestinal epithelium

**Figure 4.**
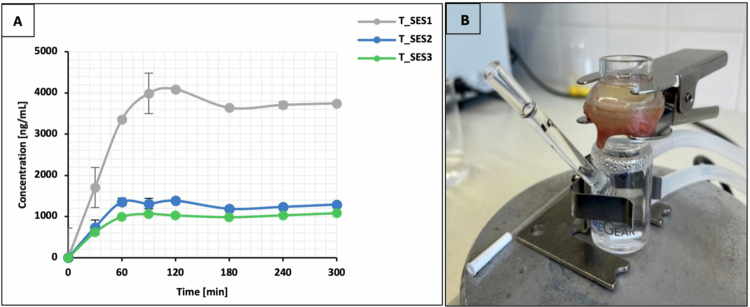
(A) Permeation of thymol-loaded SMEDDS formulations T_SES1; T_SES2; T_SES3. Data are presented as the means ± SD. (B) Franz cells with swine intestine.

To predict the absorption of the prepared phytotherapeutic formulations through the intestinal barrier, an *ex*-*vivo* permeation study was conducted using the selected SMEDDS, specifically with thymol (T_SES1, T_SES2, and T_SES3) (Nunes et al. 2016). Upon contact with aqueous media, SMEDDS formulations spontaneously form microemulsions. Studies indicate that the drug release from SEDDS occurs via diffusion (Nunes et al. 2016). Nevertheless, the speed of drug release and permeation through the mucosa depends on various factors such as particle size, surface charge, the partition coefficient of the drug between the oil phase and aqueous media, and the release-controlling effect caused by the membrane (Zaichik et al. 2018; Bernkop-Schnürch and Jalil 2018). Formulation T_SES1 exhibited the highest membrane permeation (Figure 4A). This enhanced permeation is likely attributable to its small particle size (approximately 160 nm) and the highest content of the surfactant caprylocaproyl macrogol-8 glycerides (68%), which facilitates thymol partitioning into the aqueous media. The permeation of T_SES1 was shown to be 3-fold and 3.75-fold higher than for T_SES2 and T_SES3 formulations, respectively. Intestinal mucosa contains pores in the range of 60–400 nm, with more than 50% of pores being smaller than 200 nm and around 30% being in the range of 300–400 nm (Abdulkarim et al. 2015). The lower drug permeation observed for the T_SES2 and T_SES3 formulations may, therefore, be attributed to their larger particle sizes, which exceed 250 nm. Given its superior permeation profile, *X*_SES1 was selected for further processing into a pellet dosage form and subsequent evaluation.

### 3.7. Evaluation of self-emulsifying pellets, Enprotect® capsules filling – drug content and drug release stability studies

SMEDDS possess several disadvantages when being encapsulated into capsules, such as drug leakage, instability, precipitation, and capsule aging. Solidification of self-emulsifying systems into pellet dosage forms represents an effective strategy to mitigate these issues. This approach offers numerous advantages, such as high drug loading capacity, reduced intra- and inter-subject variability in drug dissolution, enhanced stability, and improved handling properties (Wang et al. 2010; Tan et al. 2013). In addition, the pellets were prepared from materials that are safe for human consumption, biocompatible, and readily available. Together with the presumed versatility of the process for manufacturing a wider range of different products, these attributes represent important prerequisites for potential industrial-scale production (Cerveto et al. 2024).

**Figure 5.**
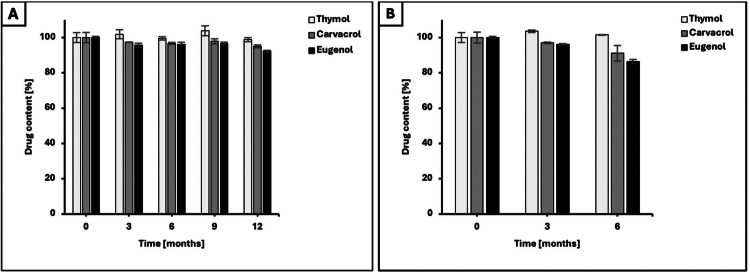
(A) Drug content stability over a 12-month period stored at 25 °C, 60% RH; (B) Drug content stability over a 6-month period stored at 40 °C, 75% RH.

Pellet cores obtained using a die plate with perforation diameters corresponding to 710–740 μm demonstrated consistently high sphericity values (0.946–0.956), indicating uniform morphology (Table S6, *Supplementary Material*). The flowability of the pellets was classified as excellent. Drug content stability studies of enteric capsules filled with self-emulsifying pellets demonstrated a stable profile over 12 months (25 °C, 60% RH) with final content of 98.8 ± 1.14%, 95.0 ± 0.79%, and 92.2 ± 0.54% for thymol, carvacrol, and eugenol, respectively (Figure 5a). Under storage conditions of 40 °C and 75% RH, the drug content was 101.5 ± 0.19%, 91.1 ± 4.42%, and 86.4 ± 1.20% for thymol, carvacrol, and eugenol, respectively, after 6 months (Figure 5b). For carvacrol and eugenol, the elevated storage conditions led to a slightly greater loss of phytotherapeutic content, suggesting that higher temperatures accelerated the volatility of these compounds. These findings highlight the importance of selecting appropriate storage conditions to preserve the stability of such formulations.

**Figure 6.**
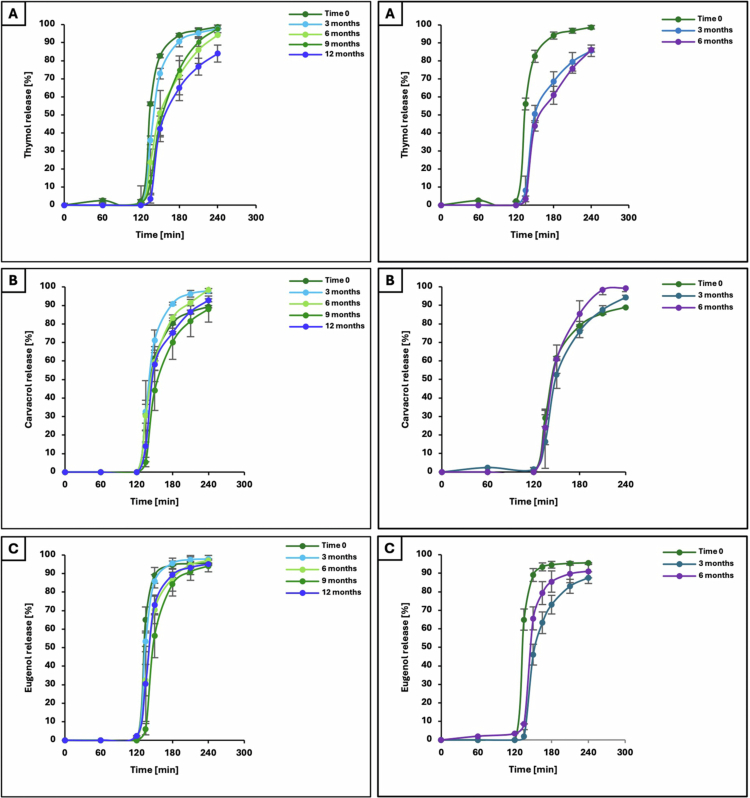
Drug release profiles (pH 1.2 changed to 6.8 after 2 h) of thymol, carvacrol, and eugenol from Capsugel® Entprotect® capsule containing 420 mg of pellets loaded with *X*_SES1 (T-thymol (A); C-carvacrol (B); E-eugenol (C)) formulations after being stored at 25 °C, 60% RH (left) and 40 °C, 75% RH (right).

The drug release profiles of all capsules filled with self-emulsifying pellets (*n* = 3) demonstrated gastro-resistant properties, with drug release remaining below 10% within the first 120 min at pH 1.2, in accordance with regulatory requirements (European Medicines Agency 2014). As shown in Figure 6, at the initial time point of the dissolution study (*t* = 0), all formulations exhibited a burst release upon release from the enteric capsule shell, with thymol, eugenol, and carvacrol leading to dissolution rates of 94.1 ± 1.96%, 91.0 ± 0.72%, and 95.5 ± 2.79%, respectively, within the first 60 minutes. Such a rapid release profile is beneficial for the intended local treatment of intestinal inflammation, as these compounds are expected to act directly on the intestinal mucosa. Rapid drug release upon reaching the intestinal environment therefore supports high local drug concentrations at the site of inflammation, whereas a sustained release profile could lead to drug distribution over a larger section of the gastrointestinal tract and consequently lower local availability at the target site.

Under mild storage conditions (25 °C, 60% RH), a decline in drug dissolution profiles was observed at 6, 9, and 12 months of stability testing. Nevertheless, all formulations achieved > 85% drug release within 120 min after a change of pH.

A more pronounced decrease in dissolution profiles was observed in formulations stored at 40 °C, 75% RH, likely due to the thermosensitive and volatile nature of the tested phytotherapeutics. However, despite these changes, all formulations still released > 85% of the loaded drug within 240 min.

### 3.8. Stability studies of enteric capsules filled with phytotherapeutic SMEDDS pellets using FTIR analysis

In the context of stability testing, the impact of SMEDDS containing volatile phytotherapeutics on the internal structure of the enteric capsules was meticulously investigated using FTIR. The ATR technique facilitates the examination of the capsule material's surface properties, enables the assessment of drug sorption into the capsule shell, and allows for the identification of structural modifications occurring throughout the testing period.

The spectra of the tested capsules were compared with the spectrum of empty, unaged enteric capsules and with the spectra of pure phytotherapeutics. This comparative analysis revealed two distinct types of spectral changes.

The first change is related to alterations in the secondary structure of the polysaccharide (hypromellose). The carbonyl vibration band, originally at 1743  cm^–1^ in untested capsules, shifts to a lower wavenumber, 1735  cm^–1^, in the spectra of all tested capsules. The band is further suppressed in the spectra of capsules tested at 40 °C. The change in the position and shape of the carbonyl band indicates changes in the secondary structure of the polysaccharide. It is confirmed by the changes in the band at 1642  cm^–1^, which is assigned to the -OH deformation vibration on the glucan ring and of adsorbed water, and at 3438  cm^–1^, which is connected with the stretching vibration of the -OH group involved in the hydrogen bonds (Figure S5, *Supplementary Material*). The decrease in the intensity of the carbonyl band is more pronounced in the spectra of the samples tested at 40 °C and is associated with a broadening of the band half-width. The position of the main bands connected with the stretching C-O-C vibration of the glycosidic linkage and in the pyranose ring (1050 and at 943  cm^–1^) and the deformation vibration of CH_2_ group (1453, 1373, and 1314  cm^–1^) remains unchanged as well as the bands in the region 3000–3750 cm^–1^linked to the stretching vibration of CH_2_ groups (Cael et al. 1975). The only changes in the polysaccharide bands are connected with the rearrangement of hydrogen bonds.

**Figure 7.**
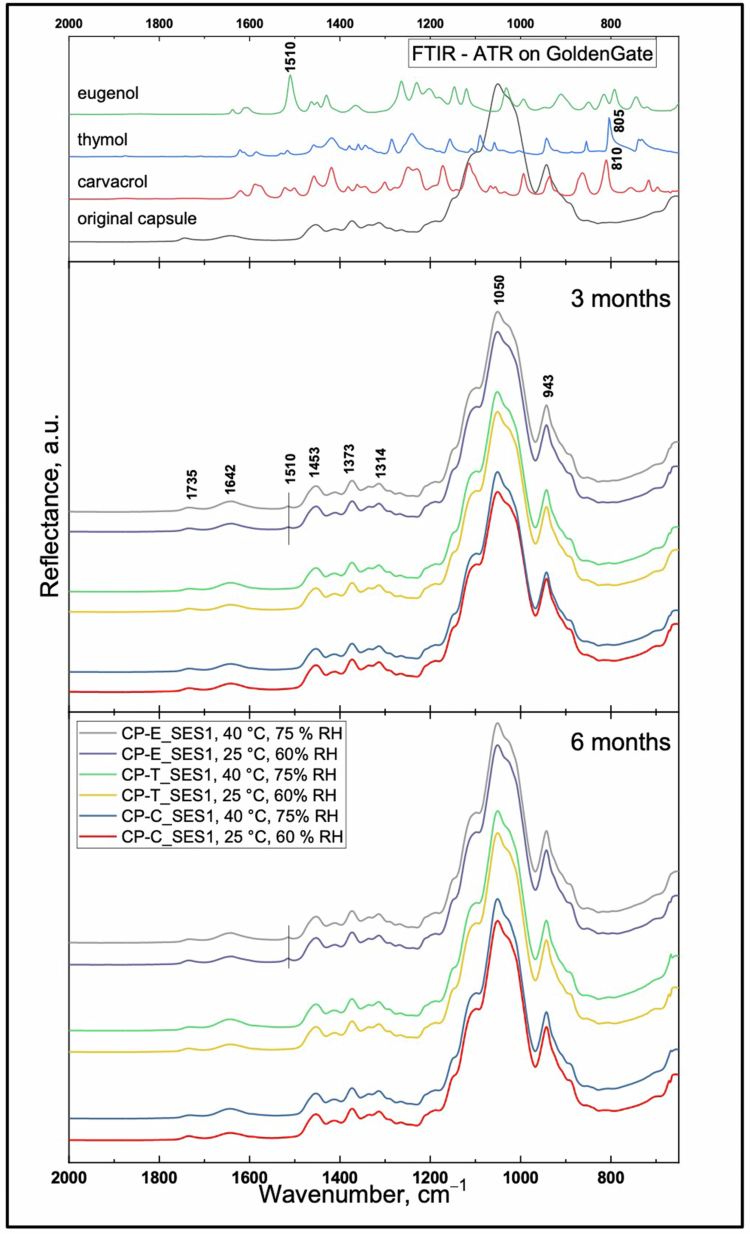
FTIR spectra of the inner layer of the capsule shell of samples CP-T_ SES1, CP-C_ SES1, and CP-E_SES1 stored under different conditions compared with the spectra of pure phytotherapeutics, respectively, and the empty Capsugel® Enprotect® capsule: after 3 months and after 6 months.

The second difference is observed in the region between 1500 and 1600  cm^–1^ in the spectrum of the capsule tested with SMEDDS loaded with eugenol. The most prominent band from the spectrum of eugenol at 1510  cm^–1^ is presented in the spectrum of the capsule after 3 and 6 months of testing, indicating sorption of eugenol into the polysaccharide matrix. The band is most pronounced after 6 months of testing at 25 °C. In contrast, the intense bands at 805 and 810  cm^–1^, characteristic of carvacrol and thymol, respectively, are absent in the spectra of tested capsules (Figure 7).

The stability testing results indicate that SMEDDS with volatile phytotherapeutics influence the internal structure of enteric capsules. FTIR analysis revealed two main spectral changes. The first involves alterations in the secondary structure of the polysaccharide (hypromellose), particularly in the carbonyl vibration band and hydrogen bonding, with more pronounced effects at elevated temperatures. The second change was observed in capsules tested with SMEDDS containing eugenol, where spectral evidence confirmed its sorption into the polysaccharide matrix over time. Conversely, no significant spectral changes were detected for carvacrol and thymol. These findings highlight the interactions between phytotherapeutic-loaded SMEDDS and capsule material, which necessitate consideration during formulation stability assessments.

### 3.9. Pharmacokinetic study in piglets

**Figure 8.**
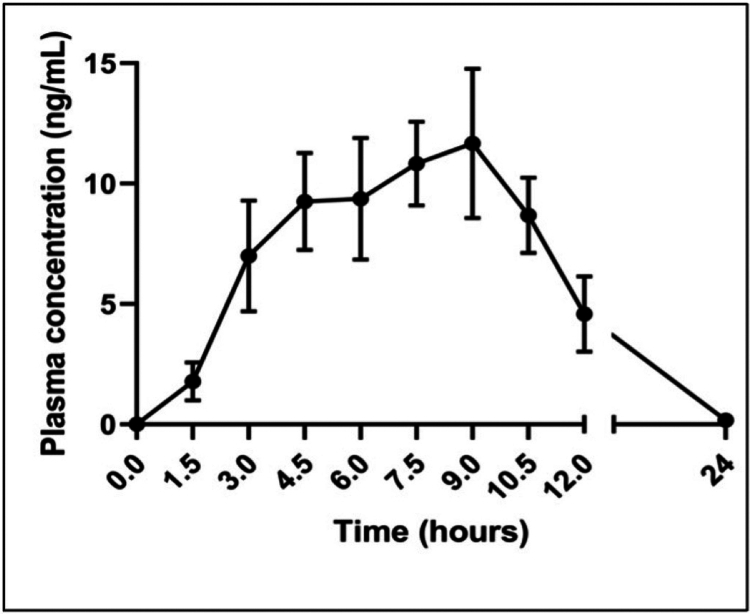
Plasma concentration–time profile of thymol following oral administration of a gastro-resistant SMEDDS-based pellet formulation to piglets (*n* = 5). Data are presented as the mean ± standard deviation.

Oral administration of the thymol-loaded self-emulsifying pellets as a filling of enteric capsules at the dose of 0.5  mg of thymol/kg to piglets resulted in a delayed peak plasma thymol concentration (C_max_ 11.67 ng/mL at 9 h post-dose) and favorable systemic exposure (AUC_0–12h of 119.8 ng·h/mL) (Figure 8). Thymol levels rose gradually to a maximum at 9 h and remained quantifiable through the 12 h sampling period, indicating sustained release and absorption. The markedly prolonged T_max_ of 9 h reflects the enteric coating effect. Thymol release was minimized in the stomach and occurred primarily in the intestines, leading to extended absorption. This controlled release profile maintained thymol plasma concentrations over time, in contrast to unformulated thymol, which is rapidly metabolized and often undetectable in plasma (Macku et al. 2022). The results demonstrate that the SMEDDS-based gastro-resistant pellet successfully achieved slow, targeted delivery of thymol with prolonged systemic availability.

These findings are consistent with recent research on advanced thymol delivery systems. Urbanová et al. (2024) showed that encapsulating thymol in Ca^2+^/Zn^2+^ crosslinked alginate–pectin hydrogels containing a self-emulsifying formulation greatly enhanced thymol absorption *in*-*vivo* (Urbanova et al. 2024). A hydrogel-based system similarly delays release until the compound reaches the higher pH environment of the intestine, improving bioavailability. Likewise, other formulations, such as SMEDDS and nanoemulsions, developed in the past recent years, have achieved superior thymol uptake compared to conventional forms. For instance, a nanoemulsion delivery of thymol was reported to penetrate gastrointestinal tissues more effectively, resulting in enhanced bioactivity relative to free thymol (Ibrahim et al. 2021). These examples underscore the benefit of formulating thymol into protected, solubilized forms to enhance its oral absorption.

Overall, the present study aligns with the growing evidence that gastro-resistance, combined with advanced delivery systems that enhance solubilization, is crucial for efficient thymol delivery. Protecting thymol from the acidic gastric milieu prevents premature dissolution and first-pass metabolism, thereby allowing more intact thymol to reach the intestine for absorption. Concurrently, the SMEDDS matrix keeps thymol dissolved in micellar form, facilitating its uptake through the intestinal wall. The delayed T_max_ and sustained plasma levels observed here confirm the success of this strategy, as the thymol was predominantly absorbed in the target intestinal region and maintained in circulation. This outcome corroborates the notion that enteric-coated formulations can overcome the low bioavailability of thymol, enabling higher systemic exposure and prolonged therapeutic effects compared to non‐protected delivery.

## 4. Conclusion

An experimental study described the development of a straightforward and effective method for delivering volatile lipophilic phytotherapeutics, known for their limited aqueous solubility, volatility, and high interaction potential, specifically to the small intestine for therapeutic and prophylactic applications. These systems involve solidifying liquid, stable SMEDDS containing individual phytopharmaceutics through an extrusion/spheronisation method to produce free-flowing pellets, which are subsequently encapsulated in commercially available gastro-resistant capsules. This approach circumvents the need for intricate coating techniques and demonstrates significant potential for broad applicability. One-year stability testing confirmed the critical requirement for storing these formulations under controlled, mild conditions to preserve drug content and gastro-resistant *in–vitro* dissolution performance, confirmed by *in–vivo* testing on animal models. Furthermore, structural examination of the internal surface of the utilized capsules revealed only minor structural alterations, predominantly observed in formulations containing eugenol. These observations enhance confidence in the study's methodology and validate the results obtained.

## Supplementary Material

Original ethics approval document MZE.pdf Original ethics approval document MZE.pdf

Supplementary material Author Checklist.pdf

Supplementary data _Cor3.docx Supplementary data _Cor3.docx

## Data Availability

The datasets generated during and/or analyzed during the current study are available from the corresponding author on reasonable request (kubovak@pharm.muni.cz).
