# Supplementary material for: Phytotherapeutics self-microemulsifying systems in pellet dosage form for enhanced intestinal drug delivery: formulation, stability, and in-vivo performance
Source: Drug Deliv. 2026 Jul 15;33(1):2702133. doi: 10.1080/10717544.2026.2702133 (PMC13374766; doi:10.1080/10717544.2026.2702133)
Supplement: Original ethics approval document MZE.pdf [file IDRD_A_2702133_SM5855.pdf]

Ministerstvo zemědělství
Odbor živočišných komodit a ochrany zvířat

Sp. zn.: MZE-3396/2023
Č. j.: MZE-20946/2023-13143

MZE-20946/2023-13143
mzedms025869341

V Praze dne: 14. 4. 2023

R O Z H O D N U T Í

Ministerstvo zemědělství, které je státním orgánem příslušným ke schvalování projektu pokusů a které rozhoduje o schválení nebo neschválení projektu pokusů z hlediska jeho souladu se zákonem č. 246/1992 Sb., na ochranu zvířat proti týrání, ve znění pozdějších předpisů (dále jen „zákon na ochranu zvířat“), a prováděcími právními předpisy vydanými k jeho provedení, a o změně, pozastavení nebo odnětí rozhodnutí o schválení projektu pokusů podle § 23 odst. 1 a § 23a odst. 1 písm. a) zákona na ochranu zvířat, v řízení zahájeném na základě žádosti podané žadatelem Výzkumný ústav veterinárního lékařství, v. v. i., zastoupeným ve správním řízení MVDr. Kateřinou Nedbalcovou, Ph.D., podle § 44 odst. 1 zákona č. 500/2004 Sb., správního řádu, ve znění pozdějších předpisů, na základě § 23a odst. 1 písm. a) zákona na ochranu zvířat ve věci schválení projektu pokusů rozhodlo takto:

Projekt pokusů č. 5/2023 „Stanovení základních farmakokinetických parametrů a biodistribuce thymolu na prasečím modelu“
(dále jen „projekt pokusů“),

podaný žadatelem Výzkumný ústav veterinárního lékařství, v. v. i., se sídlem Hudcova 296/70, 621 00 Brno (dále jen „žadatel“),

se podle § 16d a § 23a odst. 1 písm. a) zákona na ochranu zvířat z hlediska souladu se zákonem na ochranu zvířat a prováděcími právními předpisy vydanými k jeho provedení

s c h v a l u j e

pod pořadovým číslem **MZe 2422**

a to na dobu do **31. 12. 2024.**

Vedoucí projektu pokusů je doc. MVDr. Adam Novobilský, Ph.D., evidenční číslo osvědčení podle § 15d odst. 3 zákona na ochranu zvířat CZ 03433.

Projekt pokusů bude prováděn v zařízení uživatele Výzkumný ústav veterinárního lékařství, v.v.i., Hudcova 70, Brno, objekt č. 2 a objekt č. 5-1. NP.

Na projekt pokusů se nevztahuje povinnost zpětného posouzení podle § 16c zákona na ochranu zvířat.

ODŮVODNĚNÍ

Žádost o schválení projektu pokusů podle § 16a zákona na ochranu zvířat a podle § 5 vyhlášky č. 419/2012 Sb., o ochraně pokusných zvířat (dále jen „vyhláška“) byla Ministerstvu zemědělství doručena dne 18. 1. 2023. Tímto dnem bylo zahájeno správní řízení.

Výzvou ze dne 21. 3. 2023 byl žadatel vyzván k odstranění nedostatků podání a správní řízení bylo přerušeno do doby odstranění těchto nedostatků.

Doplněná žádost o schválení projektu pokusů byla Ministerstvu zemědělství doručena dne 30. 3. 2023.

Ministerstvo zemědělství posoudilo, zda je projekt pokusů v souladu se zákonem na ochranu zvířat a prováděcími právními předpisy vydanými k jeho provedení. Ministerstvo zemědělství zejména posoudilo, zda předložená žádost o schválení projektu pokusů vyhovuje kritériím podle § 16b odst. 1 zákona na ochranu zvířat. Předložená žádost kritériím stanovených zákonem na ochranu zvířat vyhověla.

Ministerstvo zemědělství v souladu s § 23a odst. 5 zákona na ochranu zvířat ustavilo rezortní odbornou komisi pro schvalování projektů pokusů a vyžádalo si v tomto správním řízení její stanovisko k předložené žádosti o schválení projektu pokusů. Rezortní odborná komise pro schvalování projektů pokusů shledala v této žádosti z hlediska jejího souladu se zákonem na ochranu zvířat a prováděcími právními předpisy vydanými k jeho provedení nedostatky, které žadatel v žádosti ze dne 30. 3. 2023 odstranil.

Na základě předložené žádosti, po provedení hodnocení projektu pokusů podle § 16b zákona na ochranu zvířat Ministerstvo zemědělství ve vyhodnocení projektu pokusů dospělo k závěru, že předložený projekt pokusů obdržel příznivé hodnocení z hledisek uvedených v § 16b zákona na ochranu zvířat. Na základě příznivého hodnocení projektu pokusů v rámci jeho hodnocení dle kritérií podle § 16b zákona na ochranu zvířat Ministerstvo zemědělství rozhodlo o schválení projektu pokusů a to na dobu do 31. 12. 2024, a to vzhledem ke splnění stanovených podmínek, které jsou v souladu se zákonem na ochranu zvířat a vyhláškou.

Podle § 16b odst. 5 zákona na ochranu zvířat, obdržel-li projekt pokusů příznivé hodnocení projektu pokusů, určí státní orgán příslušný ke schvalování projektu pokusů, zda a kdy by se měl projekt pokusů zpětně posoudit. S ohledem na požadavky ustanovení § 16c zákona na ochranu zvířat bylo ve výroku rozhodnutí stanoveno, že tento projekt pokusů nebude zpětně posuzován.

Žadatel byl vyzván k úhradě správního poplatku podle položky 75 písm. b) sazebníku poplatků – přílohy k zákonu č. 634/2004 Sb., o správních poplatcích, ve výši 1000 Kč. Tato částka byla uhrazena bankovním převodem ve stanovené lhůtě.

Žadateli bylo uděleno oprávnění k používání pokusných zvířat rozhodnutím Ministerstva zemědělství č. j. 5050/2020-MZE-18134, ze dne 23. 3. 2020. Toto rozhodnutí je platné 5 let ode dne právní moci tohoto rozhodnutí. Předložený projekt pokusů je v souladu s výše uvedeným rozhodnutím.

V souladu s § 16d odst. 5 zákona na ochranu zvířat bylo rozhodnuto v zákonem stanovené lhůtě.

Z výše uvedených důvodů bylo rozhodnuto tak, jak je uvedeno ve výroku tohoto rozhodnutí.

POUČENÍ ÚČASTNÍKŮ

Proti tomuto rozhodnutí lze podat podle § 152 odst. 1 zákona č. 500/2004 Sb., správního řádu, ve znění pozdějších předpisů, ve lhůtě 15 dnů ode dne jeho oznámení rozklad k ministru zemědělství, a to podáním učiněným u Ministerstva zemědělství, odboru živočišných komodit a ochrany zvířat.

Ing. Pavel Hakl

Digitální podpis: 14.04.2023 14:48

Ing. Pavel Hakl
ředitel odboru

Přílohy

1. VÚVeL - 5-2023 - schválená žádost o schválení projektu pokusů.pdf

Rozdělovník

Účastník řízení – Výzkumný ústav veterinárního lékařství, v. v. i., se sídlem Hudcova 296/70, 621 00 Brno, DS 3gsnh8r, zastoupeným ve správním řízení MVDr. Kateřinou Nedbalcovou, Ph.D.

Vypraveno dne
