# Supplementary material for: Phytotherapeutics self-microemulsifying systems in pellet dosage form for enhanced intestinal drug delivery: formulation, stability, and in-vivo performance
Source: Drug Deliv. 2026 Jul 15;33(1):2702133. doi: 10.1080/10717544.2026.2702133 (PMC13374766; doi:10.1080/10717544.2026.2702133)
Supplement: Supplementary data _Cor3.docx [file IDRD_A_2702133_SM5856.docx]

*Supplementary Material*

***Drug Delivery***

**Phytotherapeutics self-microemulsifying systems in pellet dosage form for enhanced intestinal drug delivery: formulation, stability and *in vivo* performance**

Gabriela Koutná^1^, Jan Kotouček^2^, Jan Macků^1^, Kateřina Kubová^1^, Martina Urbanová^3^, Larisa Janisová^3^, Ivana Sedenkova^3^, Jan Muselík^1^, Jakub Vysloužil^1^, Josef Mašek^2^, Eliška Mašková^2^, Miroslava Pavelková^1^, David Vetchý^1^ and Jiří Brus^3^

^1^ Department of Pharmaceutical Technology, Faculty of Pharmacy, Masaryk University Brno, Czech Republic; 507108@mail.muni.cz (G.K.); 451414@mail.muni.cz (J.Ma.); muselikj pharm.muni.cz (J.M.);pavelkovam@pharm.muni.cz (M.P.); vyslouzilj@pharm.muni.cz (J.V.); vetchyd@pharm.muni.cz (D.V.)

^2^ Department of Pharmacology and Toxicology, Veterinary Research Institute, Czech Republic; [jan.kotoucek@vri.cz](mailto:jan.kotoucek@vri.cz) (J.K), josef.masek@vri.cz (J.Mas.); maskova.e@vri.cz (E.M.),

^3^ Department of Structural analysis, Institute of Macromolecular Chemistry, Czech Academy of Sciences, Czech Republic; [urbanova@imc.cas.cz](mailto:urbanova@imc.cas.cz) (M.U.); [sedenkova@imc.cas.cz](mailto:sedenkova@imc.cas.cz) (I.S.); [janisova@imc.cas.cz](mailto:janisova@imc.cas.cz%60) (L.J.); [brus@imc.cas.cz](mailto:brus@imc.cas.cz) (J.B.)

**ORCiD:** G.K. 0009-0007-7725-2635; J.K. 0000-0002-3276-7228; J.Ma. 0000-0001-6234-6427; K.K. 0000-0002-4970-2084; M.U. 0000-0003-1316-6395; L.J. 0000-0002-6522-0683; I.S. 0000-0002-4128-0876; J.M. 0000-0002-4289-3885; J.V. 0000-0001-5142-3191; J.Mas. 0000-0001-6788-8487; E.M. 0000-0002-0838-7401; M.P. 0000-0001-8522-8343; D.V. 0000-0003-2476-9861; J.B. 0000-0003-2692-612X

*** Correspondence:** kubovak@pharm.muni.cz (K.K.)

**Table S1** Composition of SMEDDS preconcentrates indicated in percentage (w/w). *S_mix_ ratio refers to a ratio between caprylocaproyl macrogol-8 glycerides and diethylene glycol monoethyl ether

| **Formulation** | **Glycerol monooleate** | **Caprylocaproyl macrogol-8 glycerides** | **Diethylene glycol monoethyl ether** | **S_mix_ ratio*** |
| --- | --- | --- | --- | --- |
| **SES1** | 10.0 | 72.0 | 18.0 | 4:1 |
| **SES2** | 10.0 | 67.5 | 22.5 | 3:1 |
| **SES3** | 10.0 | 60.0 | 30.0 | 2:1 |
| **SES4** | 10.0 | 45.0 | 45.0 | 1:1 |
| **SES5** | 20.0 | 60.0 | 20.0 | 3:1 |
| **SES6** | 20.0 | 53.3 | 26.7 | 2:1 |
| **SES7** | 20.0 | 40.0 | 40.0 | 1:1 |
| **SES8** | 30.0 | 52.5 | 17.5 | 3:1 |
| **SES9** | 30.0 | 46.7 | 23.3 | 2:1 |
| **SES10** | 30.0 | 35.0 | 35.0 | 1:1 |

**Table S2** Solubility of thymol, carvacrol, and eugenol in different solvents

| **Solubility (mg/mL)** | | | |
| --- | --- | --- | --- |
| **Oils/surfactants/cosurfactants** | **Thymol** | **Carvacrol** | **Eugenol** |
| **TAG** | 693.18 | 867.61 | 842.70 |
| **Glycerol monooleate** | 693.16 | 864.43 | 954.27 |
| **Glycerol monolinoleate** | 687.37 | 1066.84 | 829.79 |
| **Caprylocaproyl macrogol-8 glycerides** | 856.44 | 978.35 | 1061.71 |
| **Propylene glycol monocaprylate** | 748.78 | 644.70 | 895.24 |
| **Oleoyl macrogol-6 glycerides** | 820.83 | 813.00 | 817.31 |
| **Diethylene glycol monoethyl ether** | 777.91 | 761.85 | 772.38 |
| **Propylene glycol** | 1015.74 | 925.19 | 868.66 |
| **Ethanol 96 %** | 779.03 | 779.38 | 641.04 |

**Table S3** Evaluation of the stability of blank formulations and formulations loaded with 5% (w/w) thymol/carvacrol/eugenol after dilution of 1:1 with Milli-Q water

|  | **Blank** | | **Drug loaded** | |
| --- | --- | --- | --- | --- |
| **Formulation** | **Appearance after mixing** | **Appearance after 24 hours** | **Appearance after mixing** | **Appearance after 24 hours** |
| **SES1** | clear | clear | clear | clear |
| **SES2** | clear | clear | clear | clear |
| **SES3** | clear | clear | clear | clear |
| **SES4** | clear | clear | turbid | separation |
| **SES5** | turbid | separation | turbid | separation |
| **SES6** | turbid | separation | turbid | separation |
| **SES7** | turbid | separation | turbid | separation |
| **SES8** | turbid | separation | turbid | separation |
| **SES9** | turbid | separation | turbid | separation |
| **SES10** | turbid | separation | turbid | separation |


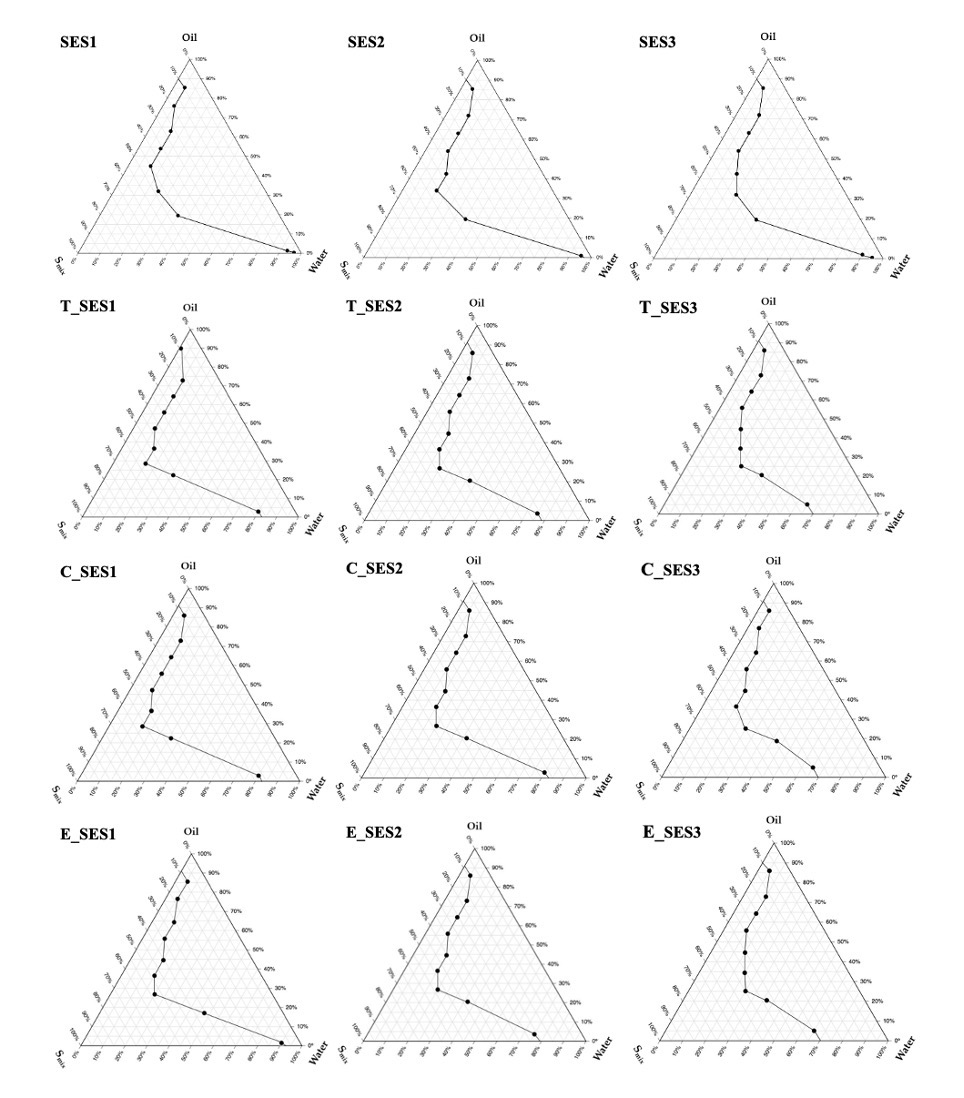


**Fig. S1** Pseudo-ternary diagrams of blank formulations – SES1 (S_mix_ 4:1); SES2 (S_mix_ 3:1); SES3 (S_mix_ 2:1). Ternary diagrams of thymol/carvacrol/ eugenol 5% (w/w) loaded formulations.

**Table S4** Droplet size and PdI evaluation of *X*_SES1, *X*_SES2, *X*_SES3 (*X* – T/C/E) formulations with progressive titration using Milli-Q water. Experiments were performed at room temperature. Indicated values are means + standard deviation (SD)

|  | **T_SES1** | | **T_SES2** | | **T_SES3** | |
| --- | --- | --- | --- | --- | --- | --- |
| **H_2_O (% w/w)** | **Z-Ave ± SD (nm)** | **PdI** | **Z-Ave ± SD (nm)** | **PdI** | **Z-Ave ± SD (nm)** | **PdI** |
| **10** | 456 ± 354 | 0.661 ± 0.187 | 1220 ± 252 | 0.983 ± 0.024 | 743 ± 260 | 0.901 ± 0.140 |
| **20** | 182 ± 21 | 0.316 ± 0.033 | 148 ± 6 | 0.367 ± 0.003 | 133 ± 18 | 0.345 ± 0.057 |
| **30** | 185 ± 1 | 0.125 ± 0.006 | 221 ± 2 | 0.331 ± 0.044 | 151 ± 1 | 0.217 ± 0.017 |
| **40** | 173 ± 1 | 0.141 ± 0.009 | 183 ± 1 | 0.209 ± 0.006 | 204 ± 1 | 0.182 ± 0.008 |
| **50** | 161 ± 1 | 0.141 ± 0.016 | 181 ± 2 | 0.169 ± 0.014 | 285 ± 11 | 0.135 ± 0.008 |
| **60** | 188 ± 0 | 0.148 ± 0.007 | 224 ± 1 | 0.158 ± 0.017 | 367 ± 8 | 0.071 ± 0.010 |
| **70** | 297 ± 2 | 0.115 ± 0.006 | 419 ± 5 | 0.075 ± 0.008 | 226 ± 62 | 0.355 ± 0.140 |
| **80** | 275 ± 58 | 0.263 ± 0.006 | 286 ± 33 | 0.253 ± 0.077 | 1210 ± 272 | 0.930 ± 0.087 |
| **Zeta-potential** | 0.031 mV | - | 0.032 mV | - | 0.012 mV | - |
|  | **C_SES1** | | **C_SES2** | | **C_SES3** | |
| **H_2_O (% w/w)** | **Z-Ave ± SD (nm)** | **PdI** | **Z-Ave ± SD (nm)** | **PdI** | **Z-Ave ± SD (nm)** | **PdI** |
| **10** | 827 ± 191 | 0.878 ± 0.087 | 490 ± 163 | 1 ± 0 | 117 ± 35 | 0.491 ± 0.129 |
| **20** | 171 ± 3 | 0.359 ± 0.060 | 170 ± 5 | 0.401 ± 0.052 | 99 ± 2 | 0.376 ± 0.026 |
| **30** | 239 ± 9 | 0.305 ± 0.036 | 147 ± 1 | 0.l56 ± 0.004 | 125 ± 1 | 0.141 ± 0.007 |
| **40** | 163 ± 1 | 0.202 ± 0.003 | 152 ± 3 | 0.200 ±0.020 | 229 ± 1 | 0.257 ± 0.007 |
| **50** | 142 ± 1 | 0.179 ± 0.008 | 145 ± 1 | 0.179 ± 0.009 | 197 ± 1 | 0.116 ± 0.014 |
| **60** | 153 ± 4 | 0.182 ± 0.009 | 173 ± 2 | 0.157 ± 0.008 | 363 ± 9 | 0.095 ± 0.021 |
| **70** | 206 ± 1 | 0.153 ± 0.012 | 277 ± 2 | 0.129 ± 0.006 | N/A | N/A |
| **80** | 398 ± 2 | 0.142 ± 0.015 | N/A | N/A | N/A | N/A |
| **Zeta-potential** | 0.022 mV | - | 0.020 mV | - | 0.012 mV | - |
|  | **E_SES1** | | **E_SES2** | | **E_SES3** | |
| **H_2_O (% w/w)** | **Z-Ave ± SD (nm)** | **PdI** | **Z-Ave ± SD (nm)** | **PdI** | **Z-Ave ± SD (nm)** | **PdI** |
| **10** | 700 ± 268 | 0.737 ± 0.139 | 1084 ± 188 | 0.958 ± 0.060 | 409 ± 199 | 0.604 ± 0.093 |
| **20** | 264 ± 69 | 0.483 ± 0.006 | 124 ± 3 | 0.392 ± 0.016 | 176 ± 25 | 0.512 ± 0.108 |
| **30** | 115 ± 1 | 0.239 ± 0.004 | 114 ± 2 | 0.298 ± 0.032 | 93 ± 2 | 0.379 ± 0.025 |
| **40** | 96 ± 1 | 0.233 ± 0.006 | 93 ± 1 | 0.222 ± 0.008 | 99 ± 0 | 0.389 ± 0.008 |
| **50** | 78 ± 0 | 0.211 ± 0.009 | 114 ± 2 | 0.328 ± 0.030 | 76 ± 2 | 0.249 ± 0.013 |
| **60** | 78 ± 0 | 0.216 ± 0.006 | 85 ± 0 | 0.199 ± 0.011 | 74 ± 1 | 0.208 ± 0.006 |
| **70** | 101 ± 1 | 0.199 ± 0.010 | 115 ± 1 | 0.167 ± 0.009 | 95 ± 1 | 0.191 ± 0.013 |
| **80** | 181 ± 5 | 0.142 ± 0.030 | 249 ± 4 | 0.119 ± 0.004 | 177 ± 2 | 0.150 ± 0.011 |
| **Zeta-potential** | 0.021 mV | - | 0.034 mV | - | 0.023 mV | - |


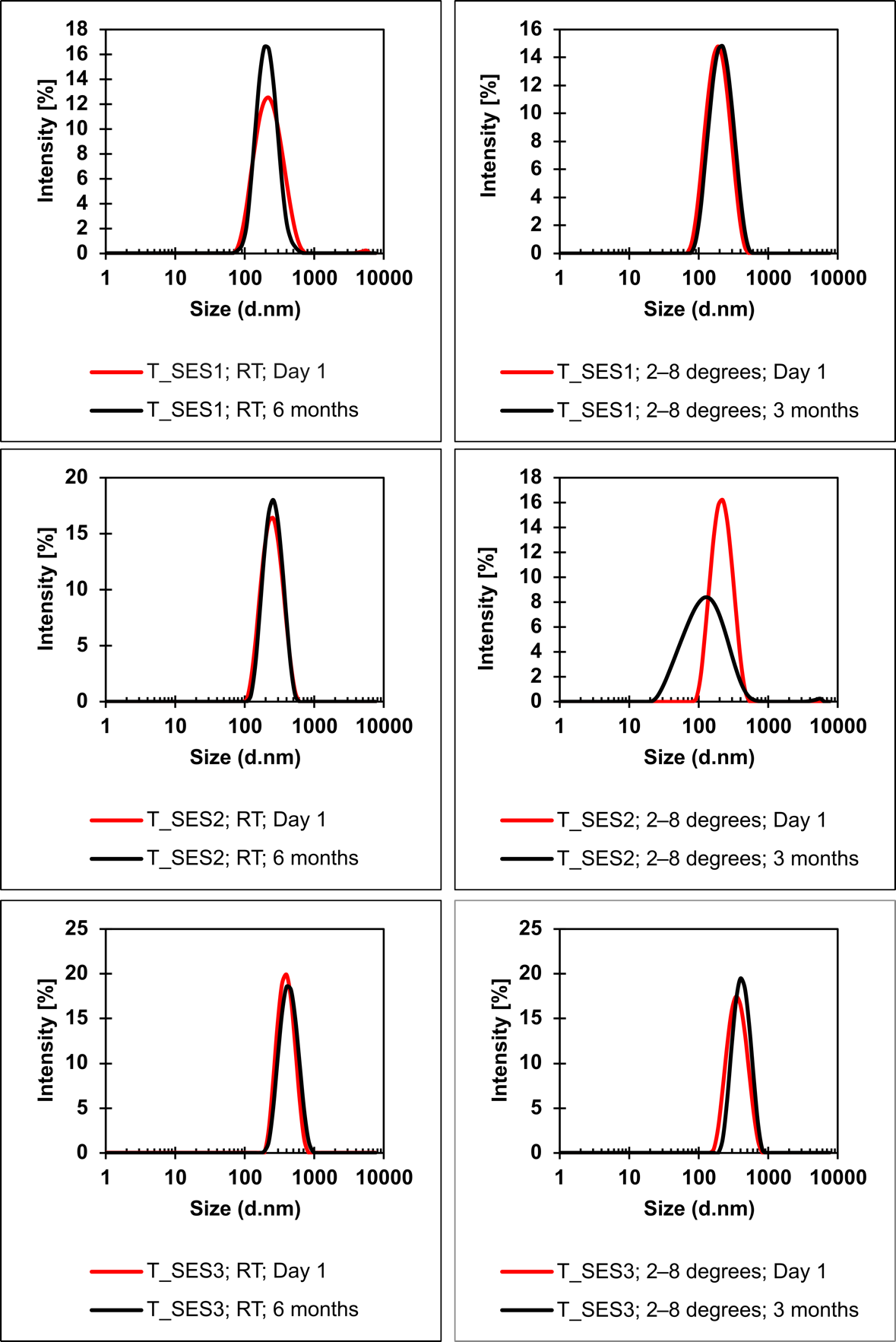


**Fig. S2** Size distribution by intensity T_SES1, T_SES2, T_SES3 systems. Samples were stored at room temperature (left column) in the fridge (right column) and and were diluted with 60% (w/w) Milli-Q water prior to each assessment.

**
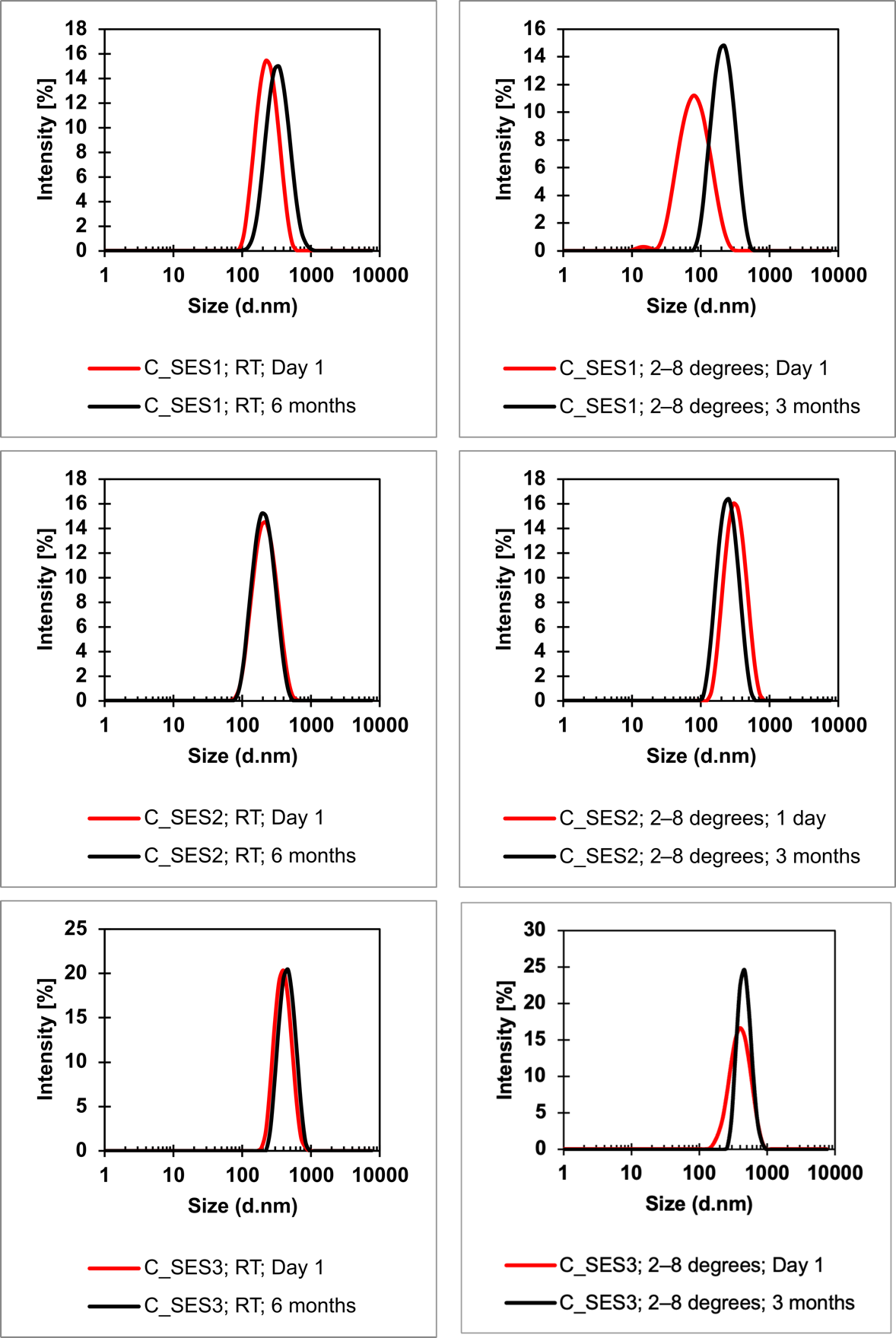
**

**Fig. S3** Size distribution by intensity T_SES1, T_SES2, T_SES3 systems. Samples were stored at room temperature (left column) in the fridge (right column) and and were diluted with 60% (w/w) Milli-Q water prior to each assessment.

**
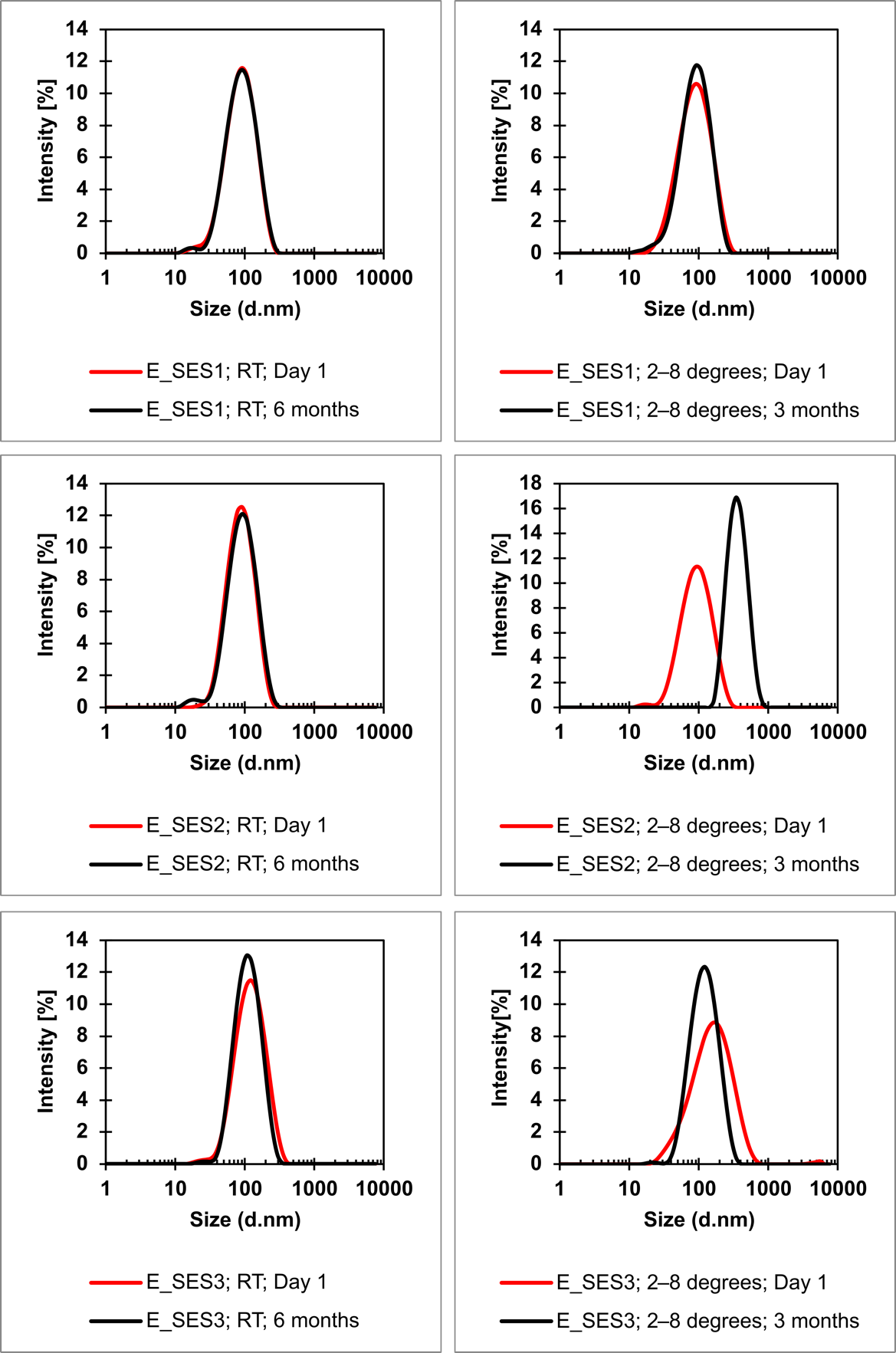
**

**Fig. S4** Size distribution by intensity E_SES1, E_SES2, E_SES3 systems. Samples were stored at room temperature (left column) in the fridge (right column) and and were diluted with 60% (w/w) Milli-Q water prior to each assessment.

**Table S5** Macroscopic evaluation of each formulation after thermodynamic stability tests exposure. *C = Grade of emulsifying proiperties (Table 2)

| Formulation | Heating-Cooling cycle | centrifuge | Freeze-Thaw Cycle | ^1^H-NMR | Disperse Grade |
| --- | --- | --- | --- | --- | --- |
| T_SES1 | Pass | Pass | Pass | No changes | C* |
| T_SES2 | Pass | Pass | Pass | Changes | C |
| T_SES3 | Pass | Pass | Pass | Changes | C |
| C_SES1 | Pass | Pass | Pass | Changes | C |
| C_SES2 | Pass | Pass | Pass | Changes | C |
| C_SES3 | Pass | Pass | Pass | Changes | C |
| E_SES1 | Pass | Pass | Pass | Changes | C |
| E_SES2 | Pass | Pass | Pass | Changes | C |
| E_SES3 | Pass | Pass | Pass | Changes | C |

**Table S6** Technological properties of self-emulsifying pellets. * According to Hausner ratio: E = excellent

| **Sample** | **Sphericity ± SD** | **Average diameter ± SD [mm]** | **Flow properties*** | **Pycnometric density ± SD (g/cm^3^)** |
| --- | --- | --- | --- | --- |
| **CP-T_SES1** | 0.956 ± 0.027 | 0.740 ± 0.070 | E | 1.4266 ± 0.0050 |
| **CP-C_SES1** | 0.951 ± 0.027 | 0.710 ± 0.080 | E | 1.4212 ± 0.0004 |
| **CP-E_SES1** | 0.946 ± 0.030 | 0.730 ± 0.080 | E | 1.4251 ± 0.0006 |


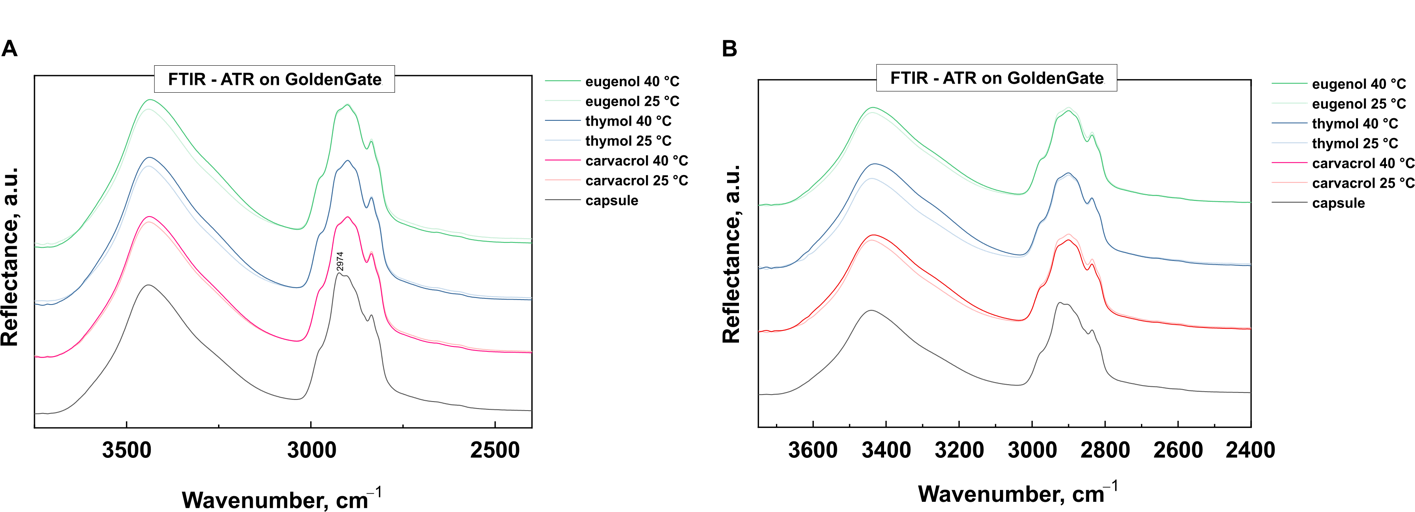


**Fig. S5** FTIR spectra - hydroxyl and aliphatic region of the inner layer of the capsule shell of samples CP-T_ SES1, CP-C_ SES1, and CP-E_ SES1 stored under different conditions compared with the spectra of pure phytoterapeutics, respectively, and the empty Capsugel^®^ Enprotect^®^ capsule: A) after 3 months and B) after 6 months
